# Leveraging Electrons for Electrochemical CO_2_ Capture Using a Hemi‐Labile Iron Complex

**DOI:** 10.1002/anie.202505723

**Published:** 2025-08-04

**Authors:** Hyowon Seo, Ying Chen, Eric Walter, Maryam Abdinejad, T. Alan Hatton

**Affiliations:** ^1^ Department of Chemical Engineering Massachusetts Institute of Technology 77 Massachusetts Avenue Cambridge MA 02139 USA; ^2^ Department of Materials Science and Chemical Engineering Stony Brook University 100 Nicolls Road Stony Brook NY 11790 USA; ^3^ Physical and Computational Sciences Directorate Pacific Northwest National Laboratory 902 Battelle Blvd Richland WA 99354 USA; ^4^ Environmental Molecular Sciences Laboratory Pacific Northwest National Laboratory 902 Battelle Blvd Richland WA 99354 USA

**Keywords:** Carbon storage, Electrochemistry, Iron, Ligand effects, Redox chemistry

## Abstract

Climate change, driven by anthropogenic carbon emissions, demands urgent action to prevent a 2050 tipping point. With CO_2_ levels at 427 ppm (50% above pre‐industrial levels), deploying energy‐efficient carbon capture technologies is crucial. Electrochemical carbon capture processes that have been touted to have the potential to meet these needs rely on the applied cell voltage, and electron utilization (CO_2_ molecules separated per electron), which has generally been asserted to have a theoretical limit of one. Here, we introduce an electron‐leveraging strategy to enhance electron utilization beyond this limit to 1.43 by employing Fe‐EDDHA, a redox‐active coordination complex having a ligand with multiple hemi‐labile coordination sites. The reversibility and robustness of the system were enabled by the efficient prevention of CO_2_ reduction upon the introduction of nicotinamide as a guardian of the iron(2+) center. The proof‐of‐concept cyclic system exhibits a minimum operational energy of 22.6 kJ_e_ mol^−1^ and an average of 63.7 kJ_e_ mol^−1^ over 29 cycles, using a simulated flue gas (15% CO_2_). Our electron‐leveraging strategy holds promise for advancing energy‐efficient electrochemical carbon capture technologies, and offers an alternative to prevalent redox potential shifting methods proposed to mitigate undesired electron transfer reactions in redox‐active materials across diverse operational conditions.

## Graphical abstract

We present an electrochemical CO_2_ capture system using coordination complex with an iron center and hemi‐labile ligands. This system achieves an electron utilization of 1.43 by leveraging oxidation‐state‐dependent ligand coordination, enabling efficient CO_2_ separation from flue gas with low energy consumption and high cyclability.

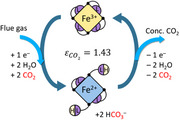

## Introduction

Climate change associated with anthropogenic carbon emissions has emerged as a foremost challenge to global stability, requiring immediate attention to prevent reaching a critical tipping point by 2050.^[^ ^1^ ^]^ Widespread deployment of carbon capture technologies with minimized energy consumption is required to effectively reduce elevated atmospheric carbon dioxide (CO_2_) levels (currently at 427 ppm, over 50% higher than pre‐industrial levels of approximately 280 ppm).^[^ ^2^, ^3^, ^4^, ^5^ ^]^ Post‐combustion carbon capture from concentrated sources, such as coal‐fired power plants, plays a critical role in the transition toward net‐zero emissions, and when combined with complementary technologies like direct air capture, it holds the potential to contribute to net‐negative emissions, thus helping to mitigate the long‐term impacts of climate change. Electrochemical carbon capture systems have emerged as a promising avenue to address the limitations inherent in established thermal carbon capture processes that rely on aqueous amine solutions.^[^ ^6^, ^7^ ^]^ These thermal systems suffer from the primary concerns of energy‐inefficient regeneration^[^ ^8^, ^9^ ^]^ and the environmental impacts stemming from the volatility of amines and the disposal of degraded amine solutions.^[^ ^10^ ^]^ In contrast, electrochemical systems offer inherent advantages such as isothermal conditions, plug‐and‐play operation, and modular scalability, with significant potential for the development of energy‐efficient carbon capture processes.^[^ ^11^, ^12^, ^13^, ^14^, ^15^ ^]^ Recent advancements in the design and utilization of non‐volatile molecular redox‐active materials for electrochemical carbon capture systems have, for instance, shown promise in achieving more environmentally benign processes.^[^ ^16^, ^17^, ^18^, ^19^, ^20^, ^21^, ^22^, ^23^, ^24^, ^25^, ^26^ ^]^

Recent research in electrochemical carbon capture systems has focused on reducing separation energy consumption (*E_sep_*, Equation 1) by optimizing the electrochemical cell potential (*E_cell_*, Equation 1) and maximizing the electron utilization ($\varepsilon_{CO_{2}}$, Equation 2). While $\varepsilon_{CO_{2}}$ typically has a theoretical limit of one,^[^ ^25^, ^27^, ^28^, ^29^, ^30^ ^]^ efforts to reduce energy consumption even further require surpassing this limit.

(1) $$\;E_{sep}=\frac{\int IVdt}{\int {\dot{n}}_{CO_{2}}dt}=\frac{n_{electron}FE_{cell}}{n_{CO_{2}}}=\frac{\;FE_{cell}}{\varepsilon_{CO_{2}}}\;$$

(2) $$\;\varepsilon_{CO_{2}}=\frac{n_{CO_{2}}}{n_{electron}}$$

where, $n_{electron}(=\int Idt/F$) is total moles electrons transferred, F is Faraday's constant (96.5 kC mol^−1^), ${\dot{n}}_{CO_{2}}$ is the instantaneous rate of release of CO_2_, and $n_{CO_{2}}$ is total moles of CO_2_ released.

**Figure 1:**
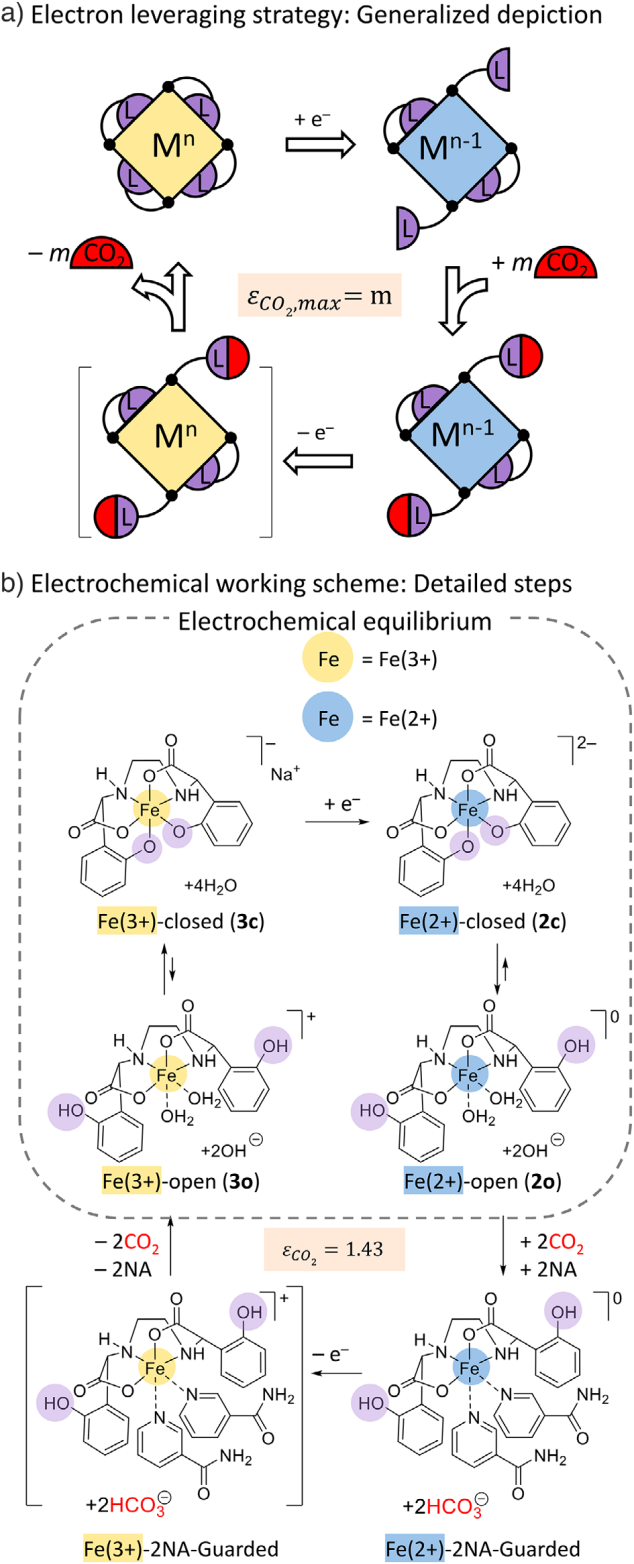
Electron‐leveraging strategy for electrochemical carbon capture using a hemi‐labile iron complex. a) Conceptual overview of the electron‐leveraging strategy using a hemi‐labile coordination complex, depicting the general interaction between the ligand and CO_2_. M: metal center, L: ligand. b) Electrochemical working scheme illustrating the carbon capture process using Fe‐EDDHA as a hemi‐labile redox‐active coordination complex in water, with NA acting as an iron center guardian. The detailed mechanism highlights the crucial role of proton transfer at the ─O─Fe and ─OH sites in facilitating the pH‐swing mediated carbon capture.

To achieve greater reductions in energy consumption, we were led to develop an electron‐leveraging strategy aimed at increasing electron utilization values beyond unity. This pursuit involves the design of redox‐active compounds with the ability to capture multiple CO_2_ molecules per electron transferred. A similar concept was proposed to achieve enhanced electron utilization via Cu(I)/Cu(II) cycling using coordination number changes in the presence of imidazole ligands, but lacked comprehensive experimental validation.^[^ ^31^ ^]^ Building upon the previously conceptualized electron‐leveraging strategy, our work introduces a significant advancement by utilizing hemi‐labile ligands. These ligands, with their coordination and proton affinities dependent on oxidation state, expand the molecular search space beyond the limitations imposed by coordination number changes, which are observed in only a few metals and specific ligand environments. Moreover, our study provides robust experimental validation of this enhanced electron‐leveraging approach, demonstrating its practical applicability in electrochemical carbon capture. Figure 1a illustrates the essential features as one specific approach within the broader electron‐leveraging strategy in the electrochemical carbon capture process, which include: 1) utilization of a coordination complex (+n oxidation state) with ligands involving a number m of hemi‐labile coordination sites, 2) occurrence of electrochemical electron transfers at the metal center, 3) alteration of the metal center's oxidation state from +n to +(*n*−1) leading to a shift in equilibrium between ligand‐closed and ligand‐open conformations of the *m* coordination sites, and 4) capture of CO_2_ under the ligand‐open conformation with a maximum electron utilization value of *m*.

To demonstrate the concept of an electron‐leveraging electrochemical carbon capture system, we selected redox‐active ferric ethylenediamine‐*di*‐(*o*‐hydroxyphenylacetate) (Fe‐EDDHA),^[^ ^32^ ^]^ a commercially available fertilizer (See Supporting Information for the purification procedure, Figures S1 and S2). In this system, depicted in Figure 1b, Fe(III)‐EDDHA‐closed (**3c**) in an aqueous electrolyte is electrochemically reduced by a single electron transfer to form Fe(II)‐EDDHA‐closed (**2c**). The Fe(II)‐EDDHA‐closed (**2c**) exists in equilibrium with the Fe(II)‐EDDHA‐open (**2o**) form. Notably, the two phenolate ligands are dissociated from the iron center and undergo protonation, leading to an increase in the pH of the electrolyte. This elevated solution pH facilitates the capture of CO_2_ from the diluted CO_2_ feed gas. Once the solution becomes saturated with CO_2_, an electrochemical oxidation results in the formation of Fe(III)‐EDDHA‐open (**3o**), which rapidly converts back to the closed conformation (**3c**), releasing protons to decrease the pH and hence drive the liberation of CO_2_.

To ensure the reversibility and efficiency of an electrochemical carbon capture system utilizing coordination complexes, it is crucial to prevent catalysis of CO_2_ reduction by the metal center.^[^ ^33^, ^34^, ^35^, ^36^ ^]^ Side reactions involving CO_2_ reduction can lead to a decrease in system efficiency by converting a portion of CO_2_ to the reduced products such as CO. The resulting impure product stream in such cases would hamper subsequent utilization or sequestration of the captured CO_2_. Additionally, potentially permanent CO poisoning^[^ ^37^, ^38^ ^]^ on the metal center can result in a reduced effective concentration of the redox‐active compound in the solution which can negatively impact carbon capture capacity, cyclability, and the overall lifetime of the system. In our system utilizing Fe‐EDDHA, which exposes open coordination sites upon reduction as proposed, there is a significant risk of CO_2_ reduction through inner‐sphere electron transfers facilitated by CO_2_ binding at these open coordination sites. To prevent this undesired electron transfer pathway, we introduced a metal center guardian rather than rely on the prevalent redox potential shifting method.^[^ ^20^, ^21^, ^26^, ^30^, ^39^ ^]^ We chose pyridine‐3‐carboxylic acid amide (or nicotinamide, NA) as pyridine, despite being neutral, causes moderately large d‐orbital splitting leading to strong bonding interaction to metal centers.^[^ ^40^, ^41^, ^42^ ^]^ This addition effectively disables CO_2_ reduction catalysis by sterically obstructing the approach of CO_2_ to the iron center (for stoichiometry, see Supporting Information Section 8).

This distinctive metal center guardian approach to mitigate undesired electron transfers at the molecular level distinguishes our work from prevalent potential‐shifting methods^[^ ^20^, ^21^, ^26^, ^30^, ^39^ ^]^ and can extend its applicability to achieving system stability beyond CO_2_ reduction, including stability toward oxygen. The critical distinction lies in the high electron density at the metal center of coordination complexes that is effectively shielded by ligands, affording the opportunity to employ suitable metal center guardians as sentinels against undesired electron transfers. In stark contrast, conjugated organic compounds like quinone and phenazine compounds have electron distribution across their conjugated structure and expose their entire aromatic structures to potential electronic interactions when encountering counterpart species, making them susceptible to electron transfer. While our system successfully demonstrates the desired metal center guarding effect under the current conditions, it is important to note that outer‐sphere electron transfer—which cannot be kinetically blocked—may still occur and can often be faster than inner‐sphere processes.

While achieving oxygen stability remains ongoing work (See Figure S6) and falls beyond the scope of this paper, the present strategy offers fundamentally distinct advantages. The reduction potentials for the closed species (**2c** and **3c**)—which determine the potential required for the electrochemical reduction step in three‐stage and four‐stage systems^[^ ^14^, ^25^ ^]^ and influence other performance metrics such as CO_2_ capture kinetics and the amount of CO_2_ separated per each electrochemical swing—remain consistent in the presence and absence of NA. Electron transfer pathway is sterically blocked (kinetic control via steric hindrance) by introduction of metal center guardian to block undesired electron transfer to oxygen. In contrast, potential shifting methods (thermodynamic control) typically alter the reduction potential to avoid undesired electron transfers to oxygen, which can negatively impact CO_2_ capture kinetics and the amount of CO_2_ separated per each electrochemical swing. Although the potential of the open forms (**2o** and **3o**) may shift with different metal center guardians,^[^ ^43^ ^]^ this primarily affects the electrochemical oxidation steps and is less of a concern for oxygen reduction reactions.

## Results and Discussion

**Figure 2:**
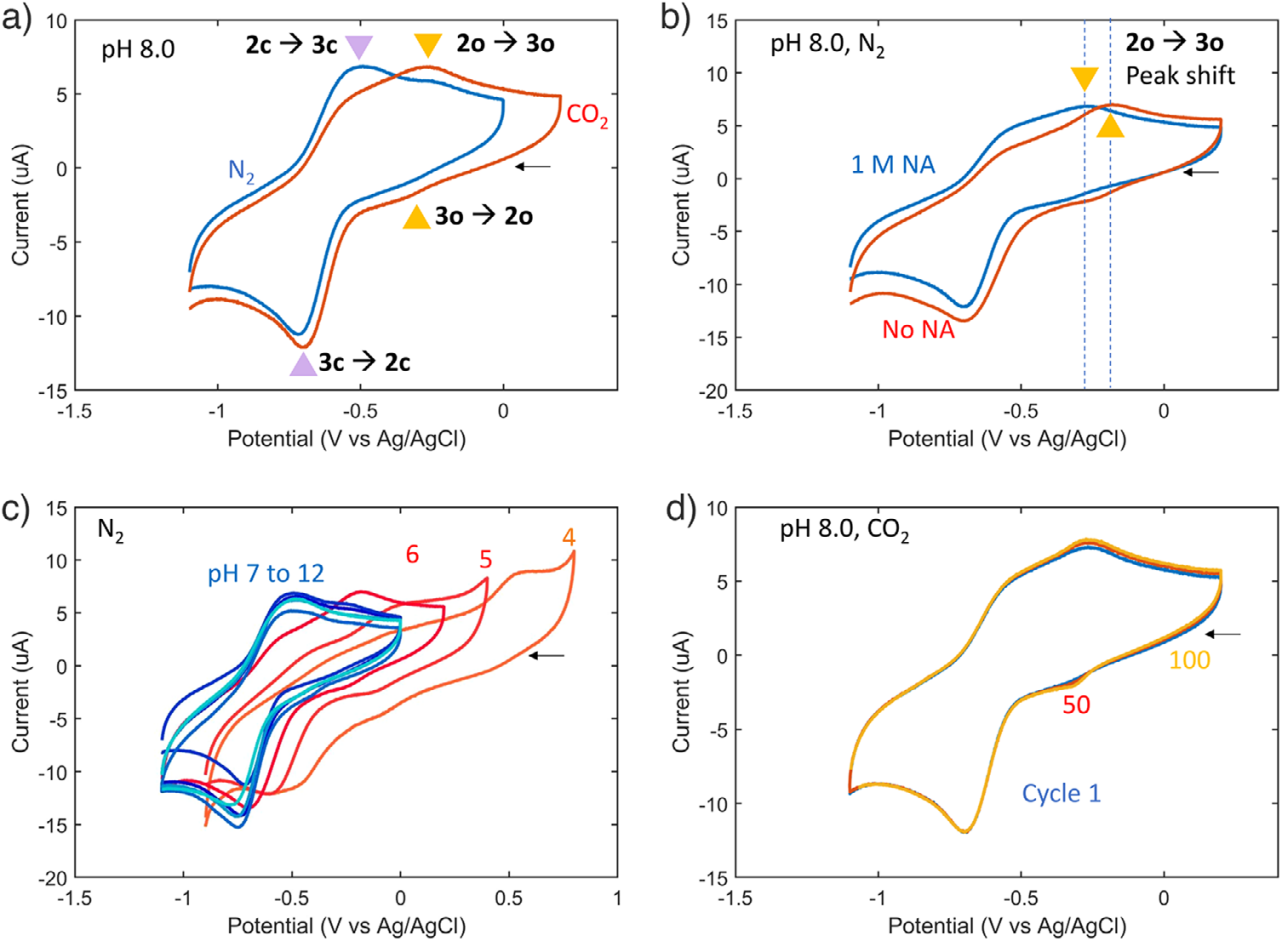
Cyclic voltammetry of Fe‐EDDHA in aqueous solutions. a) The cyclic voltammograms of 2 mM Fe‐EDDHA under nitrogen (blue curve, pH 8.0) and CO_2_ (red curve, pH 8.0) in water with 1 M NA as an iron center guardian and 0.1 M KNO_3_ as a supporting electrolyte. b) The cyclic voltammograms of 2 mM Fe‐EDDHA under nitrogen with NA (blue curve, pH 8.0) and without NA (red curve, pH 8.0) in water with 0.1 M KNO_3_ as a supporting electrolyte. c) The cyclic voltammograms of 2 mM Fe‐EDDHA at pH 4 (orange), 5 (pastel red), 6 (red), and 7–12 (blue shades, darker for pH 7 to lighter for pH 12) in 1 M NA and 0.1 M KNO_3_ solutions under N_2_. pH was adjusted by addition of potassium hydroxide before the experiments. d) 100 cyclic voltammograms of Fe‐EDDHA. Cycle 1: blue, cycle 50: red, cycle 100: yellow. All CV curves were recorded at room temperature at a scan rate of 100 mV s^−1^ with a glassy carbon working electrode (3 mm diameter) and Pt counter electrode. Potentials were recorded versus Ag/AgCl as a reference electrode. All pH was adjusted after the introduction of CO_2_ by adding 0.1 M potassium hydroxide solution or 0.1 M hydrochloric acid.

A series of cyclic voltammetry (CV) experiments were conducted to investigate the electrochemical behavior of the Fe‐EDDHA system. In Figure 2a, CV measurements are shown for the system under nitrogen (N_2_) and CO_2_ at pH 8 in an aqueous solution with 0.1 M of potassium nitrate (KNO_3_) as a supporting electrolyte and 1M of NA as an iron center guardian. Under N_2_ (blue curve), Fe‐EDDHA shows a reduction peak at −0.72 V and a quasi‐reversible oxidation peak at −0.50 V versus Ag/AgCl with another minor set of redox peaks at −0.35 V for reduction and −0.25 V for oxidation, attributed to phenolate ligands in the open conformation. Under CO_2_ (red curve), a larger oxidation peak of the **2o** was observed at −0.25 V, accompanied by a smaller oxidation peak corresponding to the closed form at −0.50 V. These results suggest equilibrium between the **2c** and **2o** forms, which can be influenced by the presence of CO_2_. Notably, regardless of the presence of CO_2_, the electrochemical reduction behavior exhibited a similar shape in the reductive scan (lower region of the curves) under both N_2_ and CO_2_ atmospheres. These findings indicate that the equilibrium between **3c** and **3o** strongly favors the closed form (**3c**) and is not significantly disrupted by the presence of CO_2_.

CV curves acquired for the Fe‐EDDHA solution, both with and without the presence of NA, an iron center guardian, are shown in Figure 2b. The curves displayed similar major peak potentials, indicating consistent electrochemical behavior of Fe‐EDDHA‐closed forms (**3c** and **2c**) regardless of the presence of NA in the aqueous solution. It is important to highlight the minor shift observed in the oxidative peak potential of **2o** from −0.33 to −0.25 V in the presence of NA, which suggests that NA stabilizes **2o** by coordinating to the iron center, leading to a positive shift in the oxidation peak within the CV curve. This observation corroborates our hypothesis regarding the blocking of the iron center by NA when **2o** is generated by electrochemical reduction.

In Figure 2c, we report a series of CV experiments under various pH conditions. Notably, identical curves were obtained within the pH range of 7 – 12. The consistent peak positions across these pH values indicate that the electron transfer process does not involve proton transfer. Notably, at pH values lower than six, we observed a significant effect of pH on the CV curves. Specifically, a larger oxidation peak for the open forms (**2o** to **3o**) was observed, and the peak shifted toward more positive potentials. These findings suggest that above a certain concentration of protons, the equilibrium between **2c** and **2o** can be shifted toward the **2o** through the protonation of the phenolate oxygen. The observed slight shifts in the reduction peak also indicate that the reduction of **3c** and **3o** is influenced by proton concentration at pH values lower than 6.

Interestingly, the CV curve obtained under CO_2_ at pH 8 (red curve, Figure 2a) aligns with the CV curve acquired under N_2_ at pH 6 (red curve, Figure 2c), i.e., the electrochemical behavior of Fe(II)‐EDDHA in the CO_2_‐saturated solution at pH 8 resembles that observed in the pH 6 solution under N_2_. This similarity can be attributed to the equilibrium shift toward **2o** induced by the presence of CO_2_. As is evident in Figure 2d, multiple CV measurements conducted over 100 cycles under CO_2_ provide evidence of the chemical stability of the hemi‐labile iron species under the given conditions.

**Figure 3:**
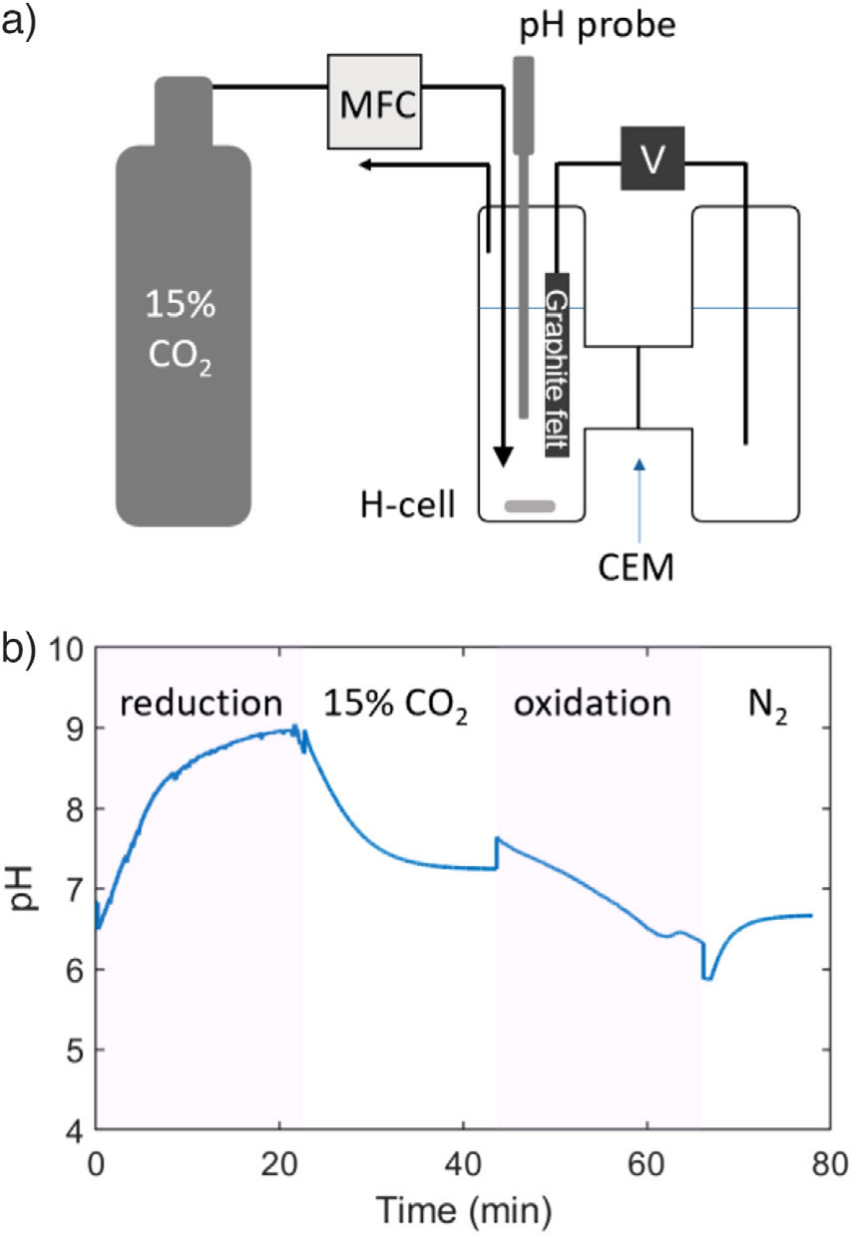
Electrochemical pH‐swing of Fe‐EDDHA system in an H‐cell. a) Schematic representation of the experimental setup for CO_2_ capture and release using Fe‐EDDHA. A 50 mM Fe‐EDDHA solution in the electrochemical H‐cell was reduced and oxidized electrochemically under a constant current mode at room temperature. The feed gas of 15% CO_2_ was supplied through a mass‐flow controller (MFC). The solution pH was monitored by a pH probe. b) pH profile of a 50 mM Fe‐EDDHA solution in water with 1 M NA and 1 M KNO_3_. Reduction under a constant current (−10 mA) for 22.5 min, 15% CO_2_ contact at a flow rate of 30 mL min^−1^ for 20 min, followed by oxidation by a constant current (10 mA) for 22.5 min, and nitrogen purging for 10 min.

A bench‐scale setup using an electrochemical H‐cell was constructed for CO_2_ capture and release in the Fe‐EDDHA redox system (Figure 3a). The system was equipped with a cation exchange membrane separating two 5 mL reaction chambers, graphite felt as a working electrode, and a stainless‐steel wire electrode for an arbitrary reaction in the counter chamber. The 4 mL reaction mixture containing 50 mM Fe(III)EDDHA (200 mmol) in water in the presence of 1 M NA as an iron center guardian and 1 M KNO_3_ as a supporting electrolyte saturated with 15% CO_2_ was reduced electrochemically in a constant current mode at 10 mA for 22.5 min (equivalent to 140 mmol of electrons transferred) to yield 70% reduction of the Fe(III)EDDHA to Fe(II)EDDHA. Then a 15% CO_2_ gas stream was introduced for 20 min at a flow rate of 30 mL/min to re‐saturate the solution, followed by anodic oxidation. The pH of the solution was monitored continuously by a pH probe throughout the CO_2_ capture and release steps (Figure 3b). During the reduction process, the pH increased from 6.8 to 9.0, which is consistent with the weak basicity of the phenolate moiety in **2o** (pKa_1_: 5.56, pKa_2_: 9.43 obtained by titration, see Supporting Figure S16; EDDHA pKa_1_: 10.2, pKa_2_: 11.744). Upon introducing the 15% CO_2_ gas stream, the solution reached a steady‐state pH of 7.3 after 20 min. During the subsequent anodic oxidation process, the pH decreased to 6.6 to complete the cycle.

**Figure 4:**
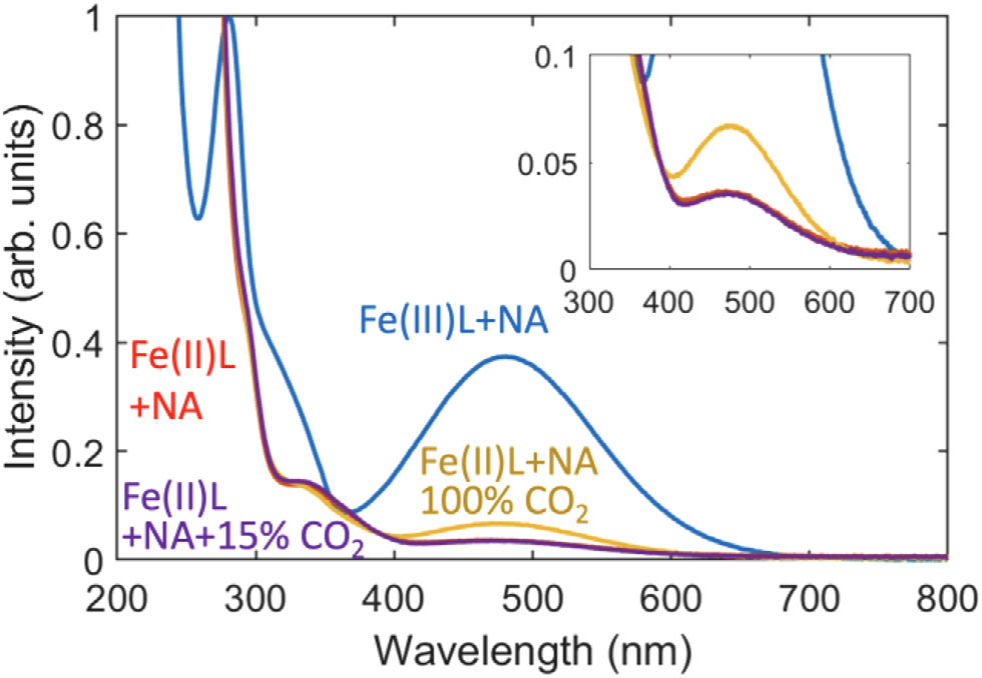
UV–vis spectra for the CO_2_ sensitivity test. The Fe(II)‐EDDHA solutions in the presence of 1 M NA (red curve, 50 mM, and 4 mL) were bubbled with 15% CO_2_ for 20 min at a flow rate of 10 mL min^−1^ (purple curve), and 100% CO_2_ for 20 min at a flow rate of 10 mL min^−1^ (yellow curve). The blue curve represents the UV–vis spectrum for Fe(III)‐EDDHA. L: EDDHA.

The stability of the Fe(II)‐EDDHA solution under a 15% CO_2_ atmosphere was assessed via UV–vis absorption spectroscopy (Figure 4). The 45 mM Fe(II)‐EDDHA solutions prepared by electrochemical reduction in the presence of 1 M NA and 1 M KNO_3_ in water were subjected to bubbling with 15% CO_2_ and 100% CO_2_ at flow rates of 10 mL min^−1^ for 20 min. The freshly prepared Fe(III)EDDHA solution showed one absorption peak at 481 nm with an intensity of 0.374; this peak intensity decreased to 0.034 after 90% reduction, and remained at this value for 20 min when contacted with 15% CO_2_. In contrast, contact with 100% CO_2_ led to a partial recovery of the peak intensity to 0.066 observed for Fe(III)EDDHA, suggesting Fe(II) oxidation to Fe(III) concurrent with CO_2_ reduction under a 100% CO_2_ atmosphere. In a set of control experiments, we observed that 60% of the Fe(III)‐EDDHA peak was recovered on the introduction of 15% CO_2_ for 20 min in the absence of NA, an iron center guardian (See Figure S5). This result supports the supposition that NA plays an essential role as an iron center guardian to avoid CO_2_ reduction via inner‐sphere electron transfer by Fe(II)‐EDDHA. While outer‐sphere electron transfer—which cannot be kinetically blocked—may still occur and can be faster than inner‐sphere processes, the desired metal center guarding effect has been clearly demonstrated under the current experimental conditions. Introduction of air for 20 min into the Fe(II)‐EDDHA solution yielded 94% recovery of the UV–vis peak at 481 nm, indicating Fe(II)‐EDDHA is rapidly oxidized back to Fe(III)‐EDDHA when in contact with oxygen (Figure S6). While the addition of NA effectively blocks electron transfer to CO_2_, its protective effect against O_2_‐induced oxidation appears limited under the current conditions.

Electron paramagnetic resonance (EPR) spectroscopy was employed to gain deeper insights into the role of NA, as illustrated in the Supporting Figure S8. Compared to the pristine solution of Fe(III)EDDHA with 47 ± 3 mM Fe(III) estimated from the EPR signal at *g*’ = 4.3, 9.1 ± 0.6 mM Fe(III), 20% of Fe(III) signal at *g*’ = 4.3 is detected after electrochemical experiments, confirming that 80% of Fe(III) is reduced to Fe(II) (Figure S8a). After 100% CO_2_ is bubbled into the Fe(II)EDDHA solutions for 5 min, the Fe(III) signal increases by 1.2‐fold in the presence of NA 11.2 ± 0.7 mM (Figure S8b) and 1.5‐fold increase in the absence of NA 13.5 ± 0.8 mM (Figure S8a), which is consistent with the UV‐vis observations in Figure 4. More importantly, in the absence of NA, the EPR signal (Figure S8c, blue curve) becomes significantly broader upon CO_2_ introduction, indicating that CO_2_ can cause a significant change in the Fe(III) coordination structure and symmetry. In contrast, in the presence of NA, the EPR signal retained its original line shape (Figure S8b, red curve), indicating that NA effectively prevents these structural changes, once again highlighting NA's role as an iron center guardian from CO_2_.

**Figure 5:**
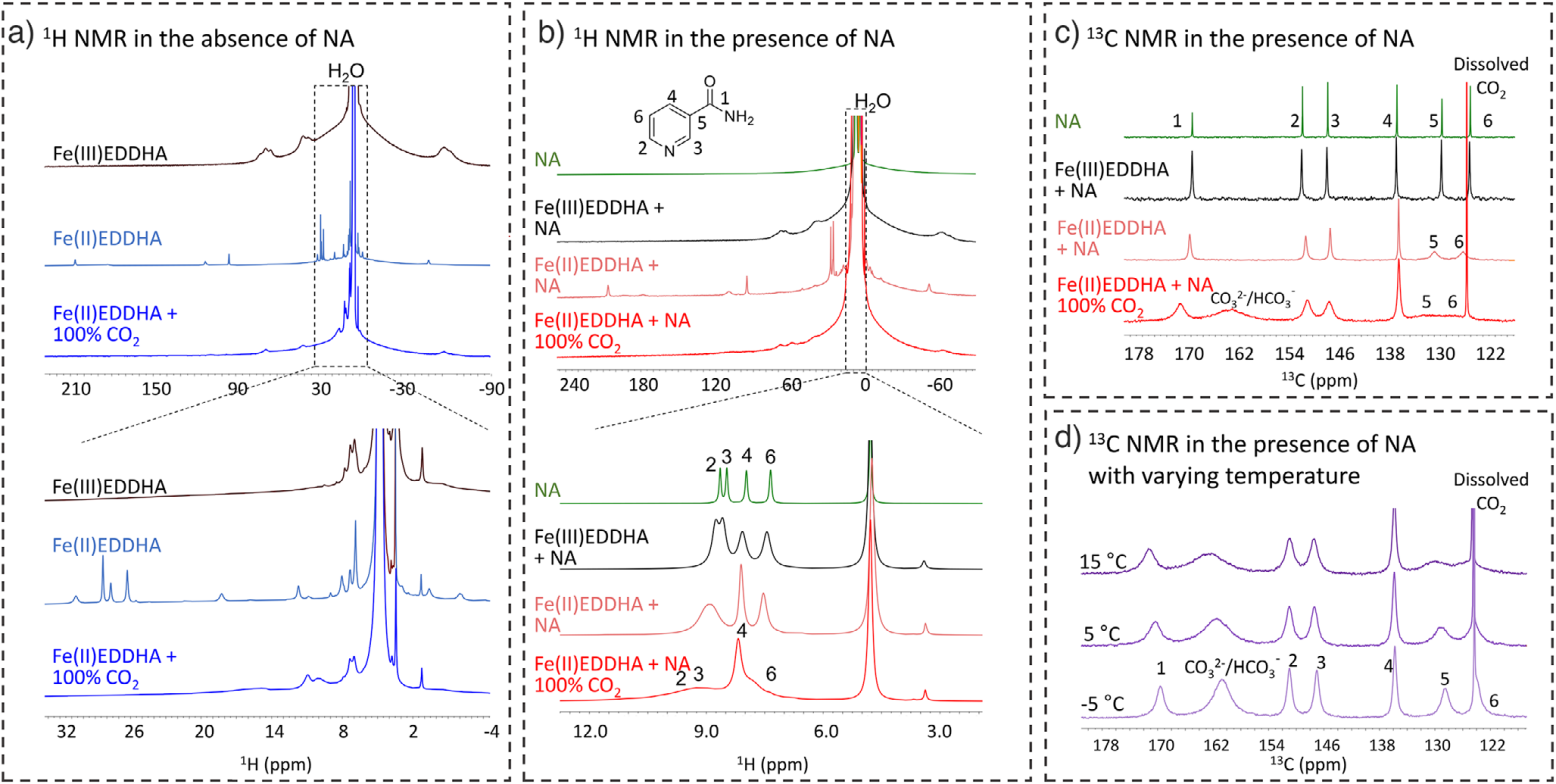
NMR studies of the stability of Fe‐EDDHA system under CO_2_. a) ^1^H NMR spectra of the 50 mM Fe‐EDDHA in 1 M KNO_3_ aqueous solutions in the absence of NA: Fe(III)‐EDDHA (black curve), Fe(II)‐EDDHA (light blue curve) and Fe(II)EDDHA with 100% CO_2_ (blue curve). A zoom‐in of the spectra between −4–32 ppm is displayed at the bottom. b) ^1^H NMR spectra of the 50 mM Fe‐EDDHA in 1 M KNO_3_ aqueous solutions in the presence of 1 M NA: NA (green curve), Fe(III)‐EDDHA + 1 M NA (black curve), Fe(II)‐EDDHA + 1 M NA (light red curve), Fe(II)‐EDDHA + 1 M NA with 100% CO_2_ (red curve). A zoom‐in of the spectra between 3–12 ppm is displayed at the bottom. c) ^13^C NMR spectra of 1 M NA (green curve), Fe(III)‐EDDHA + 1 M NA (black curve), Fe(II)‐EDDHA + 1 M NA (light red curve), and Fe(II)‐EDDHA + 1 M NA bubbled with 100% ^13^C‐enriched CO_2_ for 5 min at 25 °C (red curve). d) ^13^C NMR spectra of Fe(II)‐EDDHA + 1 M NA + 100% ^13^C‐enriched CO_2_ at varying temperatures.

Solution‐state ^1^H and ^13^C NMR were performed on a series of solutions to further understand the structural changes in FeEDDHA following electrochemical reduction, and elucidate the interactions between NA, CO_2_, and the iron center at the molecular level. In the pristine Fe(III)EDDHA solution, three sets of broad proton signals in the range of 75–60 ppm, 45–33 ppm, and −54– −70 ppm are observed and tentatively assigned to the aromatic protons, N─CH─COO, and CH_2_ protons of the EDDHA molecules coordinating to Fe(III) (Figure 5a). Some relatively sharp signals between 0 and 10 ppm can be explained by the existence of free, non‐coordinating EDDHA and of impurities in the solution. Reduction of Fe(III) to Fe(II) completely replaces these three sets of broad signals with sharp signals located at 210–170 ppm, 120–90 ppm, 30–10 ppm, and −50 ppm (Figure 5a). Although thorough assignment of ^1^H NMR signals of Fe(III)EDDHA and Fe(II)EDDHA is beyond the scope of this study, the dramatic difference in their ^1^H NMR spectra indicates the change in the coordination configurations of EDDHA with the Fe(III) and Fe(II) centers, and is consistent with the transition from Fe(III)EDDHA‐closed (**3c**) to Fe(II)EDDHA‐open (**2o**) upon electrochemical reduction. When CO_2_ was introduced to the solution, most of the sharp proton signals disappeared, and the remaining broad signals were indicative of EDDHA exchanging between Fe(II) and Fe(III) open and closed forms. Addition of 1M NA did not significantly alter the changes in EDDHA signals in the ^1^H NMR spectra (Figure 5b). However, the substantial broadening of H2, H3 and H6 signals of NA in the presence of Fe(II), especially when CO_2_ is introduced, strongly suggests the interaction between NA and Fe(II) center.

The ^13^C spectra of NA also confirm this interaction between NA and Fe(II)EDDHA (Figure 5c). While the paramagnetic Fe(III) only slightly and homogenously broadens all ^13^C signals, the diamagnetic Fe(II) shifts C5 and C6 to higher frequency by 1 ppm with even broader broadening. Introduction of CO_2_ to the Fe(II) solution further pronouncedly broadens all ^13^C signals, especially C5 and C6, and also shifts C1 to higher frequency by 1.8 ppm. These observations suggest that the entire conjugated π electron system involving the pyridine ring and the amide group coordinates to Fe(II); C5 and C6 experience the most broadening because they have the highest electron density on the pyridine ring and therefore are influenced the most by coordinating to the iron center.

The ^13^C NMR spectra of Fe(II)EDDHA in the presence of 1 M NA under the CO_2_ atmosphere, collected across a temperature range from 25 to −5 °C (Figure 5c bottom, Figure 5d), show that all ^13^C signals except for the dissolved CO_2_ signal, sharpen as the temperature decreases. While NMR signals typically broaden at lower temperatures due to reduced molecular mobility, in systems with chemical exchange between different magnetic environments, the spectra are significantly affected by exchange rates, chemical shift differences, and the fraction of molecules in each environment.^[^ ^44^ ^]^

In the case of two‐site exchange, NMR spectra exhibit a typical pattern of line broadening to a coalescence point followed by the sharping and appearance of a second set of signals, as the temperature lowers and the chemical exchange slows down.^[^ ^45^ ^]^ For instance, ^13^C spectra of 1M NA in D_2_O exhibit broadening of C2, C3, C4, and C6, while C1 and C5 signals sharpen with the appearance of new signals at higher frequencies as temperature decreases (See Supporting Figure S9). These changes are consistent with the chemical exchange between the different NA conformers observed in the crystal structures.^[^ ^46^ ^]^

A set of simulated spectra with the two‐site exchange rates varying from 5^−1^ to 0.05 s^−1^ (See Figure S10) captures several features observed in the spectral changes shown in Figure S9a. Similar to Figure S9a, the sharpening of ^13^C signals at lower temperatures in Figure 5d is a result of chemical exchange, between free NA and NA coordinating to Fe(II), with the free NA signals dominating at lower temperatures and the bound NA being undetectable even with a spectral width of 6000 ppm, indicating that the bound NA signals are too broad or beyond the detection limits of NMR. A similar sharpening pattern is observed in the ^13^C signals of C5 and C6 of Fe(II)‐EDDHA and 1 M NA solution (in the absence of CO_2_) as temperature decreases (See Supporting Figure S11). Although we cannot precisely calculate the exchange rates at this point, the clear two‐site exchange pattern suggests that the lifetime of bound NA lies within the millisecond‐to‐second range between 25 and −5 °C.^[^ ^44^ ^]^

**Figure 6:**
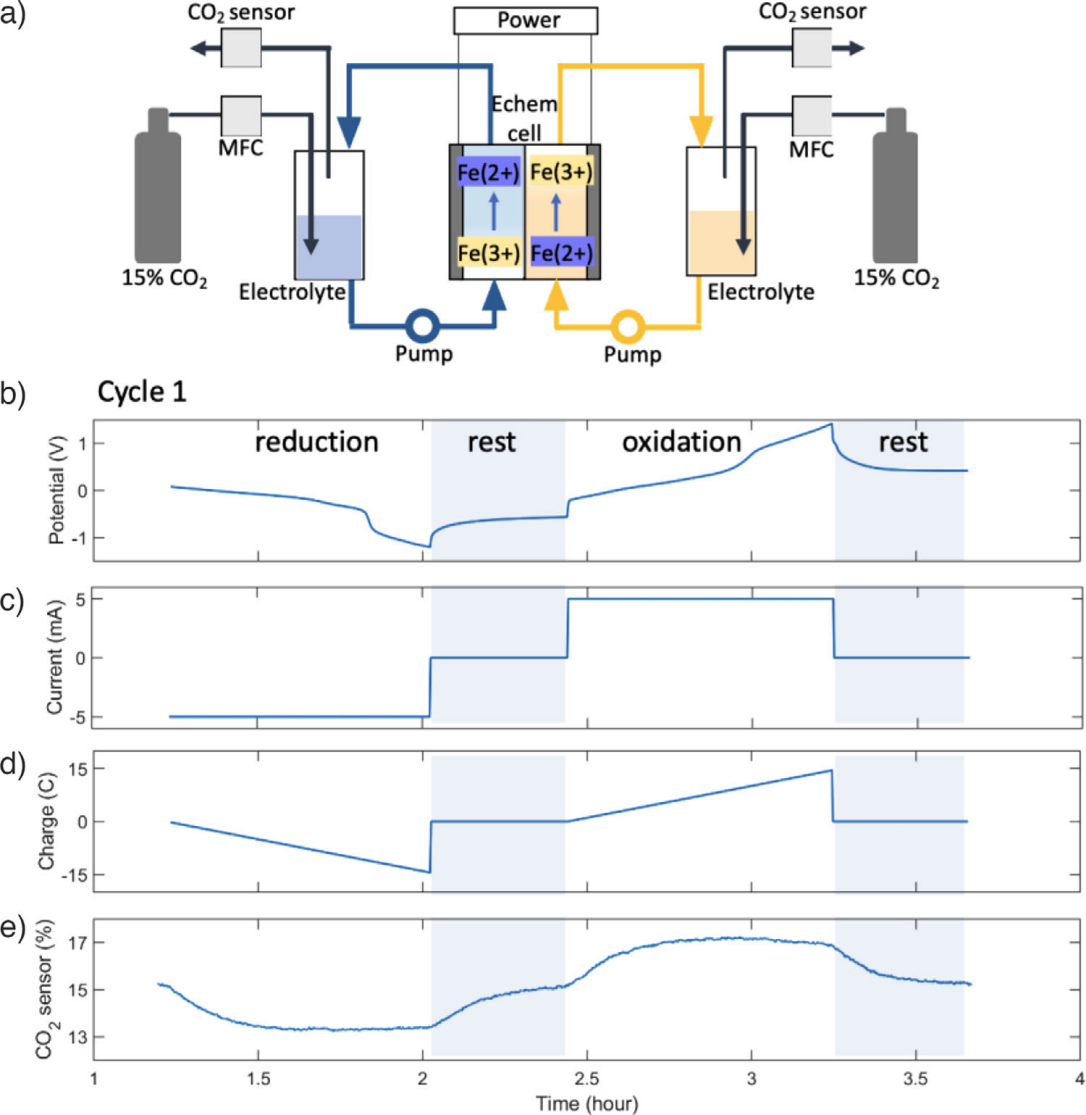
Cyclic operation of the CO_2_ capture and release using FeEDDHA redox system. a) Scheme of cyclic flow electrochemical cell using 6 mL of 50 mM FeEDDHA in 1 M NA and 1 M KNO_3_ in water for CO_2_ capture and release experiment. b) Potential. c) Current. d) Charge. e) CO_2_ sensor.

We constructed a symmetric cyclic system to process a 50 mM Fe‐EDDHA solution in the presence of 1 M NA and 1 M KNO_3_ as a supporting electrolyte in water. A schematic of the flow cell is shown in Figure 6a (See Section 3 for detailed setup information and Figure S3 for a visual representation of the setup). The flow cell structure included graphite‐felt electrodes in both chambers and a cation exchange membrane that divided the cell into the cathodic and anodic chambers and enabled the pH difference between them to be maintained. The system was equipped with two reservoirs, one for catholyte and one for anolyte, which were alternated by cycling. Both chambers were equipped with 15% CO_2_ inlet and outlet streams with output gas concentration determined by a CO_2_ sensor. We utilized 50% of the capacity of the system to minimize undesired side reactions under the constant current mode of operation at 5 mA with 15% CO_2_ at a flow rate of 5.3 mL min^−1^. The first cycle of the system is illustrated in Figure 6b–e. During this cycle, the cell potential was recorded as in Figure 6b under the application of negative and positive 5 mA currents (Figure 6c). When a negative potential was applied, the working electrode side of the cell performed reduction of the Fe(III)‐EDDHA resulting in the alkalization of the aqueous solution and the capture of CO_2_, indicated by the decrease in the CO_2_ percentage of the output stream (Figure 6e). On the counter electrode side, oxidation of Fe(II)‐EDDHA occurred, releasing CO_2_. Conversely, when a positive potential was applied, the working electrode side performed oxidation of the Fe(II)‐EDDHA solution, causing the solution to become acidic and release CO_2_, as indicated by the increased CO_2_ percentage of the output gas stream (Figure 6e). The achieved operational energy consumption was determined to be 22.6 kJ_e_ mol^−1^ of CO_2_, with an average electron utilization of 1.43.

**Figure 7:**
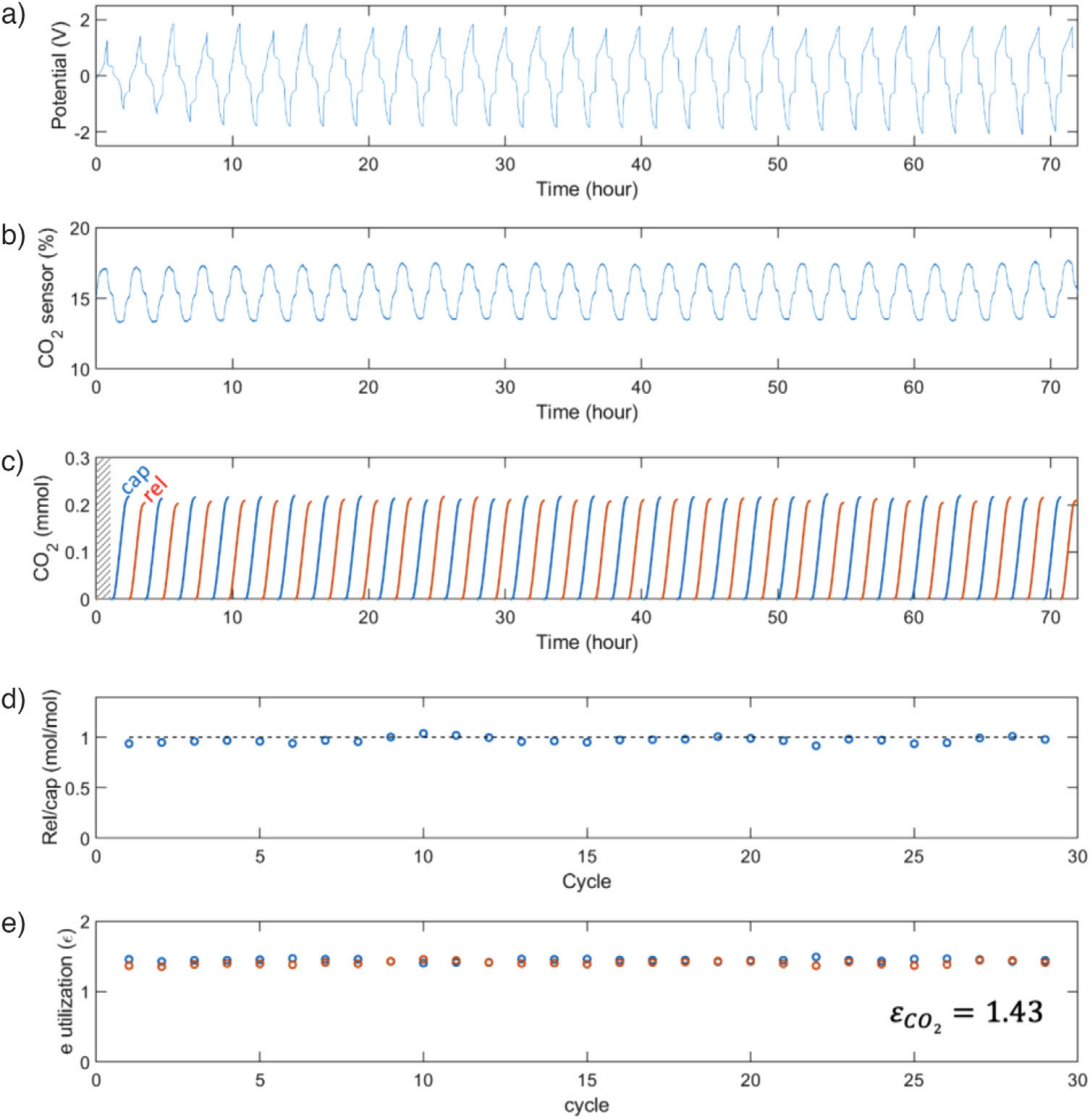
Multiple cycles demonstration of CO_2_ capture and release using Fe‐EDDHA redox system. a) Cell voltage for 29 cycles at a constant current mode of 5 mA. b) CO_2_ sensor for 29 cycles. c) Captured (blue curve) and released (red curve) CO_2_ amount using 15% CO_2_ with flow rate of 5.3 mL min^−1^. d) Ratio of released CO_2_ amount over captured amount. e) Electron utilization (captured: blue, released: red).

The cyclic experiments conducted over 29 cycles at a constant current mode of 5 mA are described in Figure 7. An average electron utilization of 1.43 was achieved over 29 cycles, along with 0.94 V of average voltage, resulting in an operational energy of 63.7 ± 1.0 kJ_e_ mol^−1^ (See Supporting Information Section 4 for calculations). These cyclic experiments provided robust support for our electron‐leveraging strategy, as they demonstrate electron utilization values exceeding unity, thereby reducing operational energy consumption with a 15% CO_2_ feed. The ratio between the released CO_2_ amount and the captured amount of one was maintained over multiple cycles (Figure 7d). Indicating that the reversible capture and release of CO_2_ occurred without side reactions such as CO_2_ reduction. Following the cyclic experiments, UV–vis measurements of the solutions confirmed the stability of the Fe‐EDDHA species without significant decrease in concentrations (See Figure S7). However, it should be noted that the system displayed sensitivity to oxygen, presumably leading to cell voltage increases between cycles. Additional cyclic experiment for 14 cycles (See Figure S15) with assembly of cell components in a glovebox for extensive air removal presented stable and reversible CO_2_ capture and release with an electron utilization value of 1.40, at decreased cell voltage average of 0.39 V, corresponding to 23.9 kJ_e_ mol^−1^ of energy requirement. Inter‐cyclic potential increases suggest potential air contact during circulation of solutions through multiple cycles.

The cyclic experiment presented here is a conceptual demonstration aimed at assessing the stability and cyclability of the system during repeated redox cycles. The energy calculations derived from this experiment provide insight into the electrochemical energy consumed per mole of CO_2_ separated under the specific experimental conditions and should be interpreted as conceptual estimates (See Supporting Information Section 10 for comparison to other carbon capture systems utilizing redox‐active molecules and benchmark systems). It is important to note that the current experimental setup, which flows 15% CO_2_ through both the anolyte and catholyte, does not fully represent the conditions necessary for producing a pure CO_2_ stream. Furthermore, these energy estimates may be underestimated due to the low separation extent observed in the cyclic system under the current experimental conditions. A more realistic assessment of the energy demand to achieve pure CO_2_ output is necessary for practical applications, as optimizing cell design and operating conditions will be essential for reaching 100% CO_2_ concentration.

## Conclusion

In conclusion, our study introduces the use of hemi‐labile ligands, which exhibit unique coordination behaviors dependent on the oxidation state of the metal center, thereby broadening and enhancing the applicability of the electron‐leveraging strategy. This approach expands the potential for optimization and increases efficiency in electrochemical carbon capture systems. We demonstrated this strategy by employing Fe‐EDDHA with dissociation of two phenolates upon a one‐electron reduction at the metal center. The introduction of NA as an iron center guardian effectively prevented CO_2_ reduction at the metal center, ensuring system reversibility, efficiency and stability. Our proposed mechanism of electron leveraging and metal center blocking at the molecular level is supported by a combination of UV–vis, NMR, and EPR analyses. We demonstrated the robustness, stability, and reversibility of the cyclic electrochemical cell operation using homogeneous aqueous solutions and a 15% CO_2_ feed. The minimum operational energy consumption achieved using a proof‐of‐concept cyclic experiment was 22.6 kJ_e_ mol^−1^ of CO_2_, with an average of 63.7 ± 1.0 kJ_e_ mol^−1^ for 29 cycles, utilizing an average electron utilization value of 1.43, exceeding the previous theoretical limit of one.

Furthermore, we anticipate that the present work will stimulate further exploration of this electron‐leveraging strategy by utilization of reversible hemi‐labile coordination complexes as redox‐active materials in electrochemical carbon capture. This exploration may extend beyond the current 2X leveraged system to more advanced 3X or even 4X leveraged systems. In addition, the redox center blocking strategy holds potential for efficiently achieving redox‐active material stability from undesired electron transfer reactions under various operational conditions, particularly in the presence of oxygen. These research endeavors aim to achieve a more substantial reduction in energy consumption and establish electrochemical carbon capture processes that are robust, efficient, durable, and scalable toward a more sustainable future.

## Conflict of Interests

The authors declare no conflict of interest.

## Supporting information



Supporting Information

## Data Availability

The data that support the findings of this study are available from the corresponding author upon reasonable request.

## References

[1] Intergovernmental Panel On Climate Change (Ipcc), Climate Change 2022 , Impacts, Adaptation and Vulnerability: Working Group II Contribution to the Sixth Assessment Report of the Intergovernmental Panel on Climate Change, Cambridge University Press, Cambridge, UK 2023.

[2] N. US Department of Commerce , “Global Monitoring Laboratory – Carbon Cycle Greenhouse Gases,” can be found under https://gml.noaa.gov/ccgg/trends/monthly.html, (accessed 2025–03‐07).

[3] B. S. Koelbl , M. A. Van Den Broek , A. P. C. Faaij , D. P. Van Vuuren , Climatic Change 2014, 123, 461–476.

[4] M. Bui , C. S. Adjiman , A. Bardow , E. J. Anthony , A. Boston , S. Brown , P. S. Fennell , S. Fuss , A. Galindo , L. A. Hackett , J. P. Hallett , H. J. Herzog , G. Jackson , J. Kemper , S. Krevor , G. C. Maitland , M. Matuszewski , I. S. Metcalfe , C. Petit , G. Puxty , J. Reimer , D. M. Reiner , E. S. Rubin , S. A. Scott , N. Shah , B. Smit , J. P. M. Trusler , P. Webley , J. Wilcox , N. M. Dowell , Energy Environ. Sci. 2018, 11, 1062–1176.

[5] A. Dubey , A. Arora , J. Cleaner Prod. 2022, 373, 133932.

[6] G. T. Rochelle , Science 2009, 325, 1652–1654. 19779188 10.1126/science.1176731

[7] P. Panja , B. McPherson , M. Deo , CCST 2022, 3, 100041.

[8] T. N. G. Borhani , A. Azarpour , V. Akbari , S. R. Wan Alwi , Z. A. Manan , Int. J. Greenh. Gas Control 2015, 41, 142–162.

[9] M. Fasihi , O. Efimova , C. Breyer , J. Cleaner Prod. 2019, 224, 957–980.

[10] G. T. Rochelle , Curr. Opin. Chem. Eng. 2012, 1, 183–190.

[11] M. Rahimi , A. Khurram , T. A. Hatton , B. Gallant , Soc. Rev. 2022, 51, 8676–8695. 10.1039/d2cs00443g 36177895

[12] A. M. Zito , L. E. Clarke , J. M. Barlow , D. Bím , Z. Zhang , K. M. Ripley , C. J. Li , A. Kummeth , M. E. Leonard , A. N. Alexandrova , F. R. Brushett , J. Y. Yang , Chem. Rev. 2023, 123, 8069–8098. 37343385 10.1021/acs.chemrev.2c00681

[13] Y. Liu , É. Lucas , I. Sullivan , X. Li , C. Xiang , iScience 25, 105153. 10.1016/j.isci.2022.105153 PMC9529983 36204263

[14] H. Seo , MRS Commun. 2023, 13, 994–1008. 10.1557/s43579-023-00454-y.

[15] H. Seo , M. P. Nitzsche , T. A. Hatton , Acc. Chem. Res. 2023, 56, 3153–3164. 37949611 10.1021/acs.accounts.3c00430

[16] S. Jin , M. Wu , R. G. Gordon , M. J. Aziz , D. G. Kwabi , Energy Environ. Sci. 2020, 13, 3706–3722.

[17] S. Pang , S. Jin , F. Yang , M. Alberts , L. Li , D. Xi , R. G. Gordon , P. Wang , M. J. Aziz , Y. Ji , Nat. Energy 2023, 8, 1126–1136.

[18] H. Seo , T. A. Hatton , Nat. Commun. 2023, 14, 313. 36658126 10.1038/s41467-023-35866-w PMC9852473

[19] H. Seo , M. Rahimi , T. A. Hatton , J. Am. Chem. Soc. 2022, 144, 2164–2170. 35020393 10.1021/jacs.1c10656

[20] X. Li , X. Zhao , Y. Liu , T. A. Hatton , Y. Liu , Nat. Energy 2022, 7, 1065–1075.

[21] J. M. Barlow , J. Y. Yang , J. Am. Chem. Soc. 2022, 144, 14161–14169. 35881543 10.1021/jacs.2c04044

[22] H. Xie , W. Jiang , T. Liu , Y. Wu , Y. Wang , B. Chen , D. Niu , B. Liang , Cell Rep. Phys. Sci. 2020, 1, 100046.

[23] H. Xie , Y. Wu , T. Liu , F. Wang , B. Chen , B. Liang , Appl. Energy 2020, 259, 114119.

[24] K. M. Diederichsen , Y. Liu , N. Ozbek , H. Seo , T. A. Hatton , Joule 2022, 6, 221–239.

[25] J. M. Barlow , L. E. Clarke , Z. Zhang , D. Bím , K. M. Ripley , A. Zito , F. R. Brushett , A. N. Alexandrova , J. Y. Yang , Chem. Soc. Rev. 2022, 51, 8415–8433. 36128984 10.1039/d2cs00367h

[26] F. Simeon , M. C. Stern , K. M. Diederichsen , Y. Liu , H. J. Herzog , T. A. Hatton , J. Phys. Chem. C 2022, 126, 1389–1399.

[27] M. Massen‐Hane , K. M. Diederichsen , T. A. Hatton , Nat Chem Eng 2024, 1, 35–44.

[28] R. Sharifian , R. M. Wagterveld , I. A. Digdaya , C. Xiang , D. A. Vermaas , Energy Environ. Sci. 2021, 14, 781–814.

[29] J. H. Rheinhardt , P. Singh , P. Tarakeshwar , D. A. Buttry , ACS Energy Lett. 2017, 2, 454–461.

[30] Y. Jing , K. Amini , D. Xi , S. Jin , A. Alfaraidi , E. Kerr , R. Gordon , M. Aziz , ACS Energy Lett., 2024, 9, 3526–3535.

[31] J. Boualavong , C. A. Gorski , ACS EST Eng. 2021, 1, 1084–1093.

[32] P. Schröder , D. Obendorf , T. Bechtold , ChemElectroChem 2019, 6, 3311–3318.

[33] S. Gonell , A. J. M. Miller , in Advances in Organometallic Chemistry, Academic Press (Elsevier), Cambridge, MA, USA 2018, 70, pp. 1–69.

[34] R. Bonetto , F. Crisanti , A. Sartorel , ACS Omega 2020, 5, 21309–21319. 32905319 10.1021/acsomega.0c02786 PMC7469117

[35] I. Bhugun , D. Lexa , J.‐M. Savéant , J. Am. Chem. Soc. 1996, 118, 1769–1776.

[36] C. Costentin , M. Robert , J.‐M. Savéant , A. Tatin , Proc. Natl. Acad. Sci. USA 2015, 112, 6882–6886. 26038542 10.1073/pnas.1507063112 PMC4460501

[37] C. Cometto , L. Chen , P.‐K. Lo , Z. Guo , K.‐C. Lau , E. Anxolabéhère‐Mallart , C. Fave , T.‐C. Lau , M. Robert , ACS Catal. 2018, 8, 3411–3417.

[38] S. Fernández , F. Franco , C. Casadevall , V. Martin‐Diaconescu , J. M. Luis , J. Lloret‐Fillol , J. Am. Chem. Soc. 2020, 142, 120–133. 31820956 10.1021/jacs.9b06633

[39] X. Li , X. Zhao , L. Zhang , A. Mathur , Y. Xu , Z. Fang , L. Gu , Y. Liu , Y. Liu , Nat. Commun. 2024, 15, 1175. 38331931 10.1038/s41467-024-45410-z PMC10853560

[40] S. Pal , in Pyridine (Ed.: P.P. Pandey ), InTech, London, UK 2018, pp. 3526–3535.

[41] S. A. Cotton , J. Coord. Chem. 2018, 71, 3415–3443.

[42] A. Santoro , L. J. Kershaw Cook , R. Kulmaczewski , S. A. Barrett , O. Cespedes , M. A. Halcrow , Inorg. Chem. 2015, 54, 682–693. 25563430 10.1021/ic502726q

[43] A. Paul , R. Borrelli , H. Bouyanfif , S. Gottis , F. Sauvage , ACS Omega 2019, 4, 14780–14789. 31552317 10.1021/acsomega.9b01341 PMC6751539

[44] A. D. Bain , Prog. Nucl. Magn. Reson. Spectrosc. 2003, 43, 63–103.

[45] B. E. Mann , Prog. Nucl. Magn. Reson. Spectrosc. 1977, 11, 95–114.

[46] W. B. Wright , G. S. D. King , Acta Crystallogr. 1954, 7, 283–288.

