# Supplementary material for: Leveraging Electrons for Electrochemical CO2 Capture Using a Hemi‐Labile Iron Complex
Source: Angew Chem Int Ed Engl. 2025 Aug 4;64(38):e202505723. doi: 10.1002/anie.202505723 (PMC12435444; doi:10.1002/anie.202505723)
Supplement: Supplementary file 1 — Supporting Information [file ANIE-64-e202505723-s001.docx]

Supporting Information

**Leveraging electrons for electrochemical CO_2_ capture using a hemi-labile iron complex**

Hyowon Seo^1,2^*, Ying Chen^3^, Eric Walter^4^, Maryam Abdinejad^1^, and T. Alan Hatton^1^*

^1^Department of Chemical Engineering, Massachusetts Institute of Technology, Cambridge, MA 02139,

USA

^2^Department of Materials Science and Chemical Engineering, Stony Brook University, Stony Brook, New York 11794, USA

^3^Physical and Computational Sciences Directorate, Pacific Northwest National Laboratory, Richland, Washington 99354, USA.

^4^Environmental Molecular Sciences Laboratory, Pacific Northwest National Laboratory, Richland, WA 99354, USA

Email: [hyowon.seo@stonybrook.edu](mailto:hyowon.seo@stonybrook.edu); tahatton@mit.edu

**Contents**

1. General Information 3
2. Procedures for Electrochemical CO_2_ Capture and Release 6
3. Setup of FeEDDHA Redox System for the Electrochemical CO_2_ Capture and Release 8
4. Energy Calculation 9
5. Additional UV-Vis Absorption Spectroscopy Data 10
6. Additional NMR/EPR Spectroscopy Data 13
7. Additional Cyclic Flow Experiment Results 20
8. Additional Mass Spectroscopy Experiment 21
9. Determination of pKa Values for Fe-EDDHA via Titration Experiments 22
10. Bjerrum plot under 15% CO_2_. 23
11. Summary of Selected Electrochemical Carbon Capture Systems 24
12. References 26

**1. General Information**

**1.1 Material**

Commercially available chemicals were purchased from Sigma-Aldrich Chemical Company (Milwaukee, WI) and were used as received. FeEDDHA was purchased from Grow More (Gardena, CA). Ag/AgCl reference electrodes were purchased through BASi (West Lafayette, IN). CO_2_ cylinders (1, 4, 15, and 100% balanced by nitrogen) were purchased from Airgas (Radnor Township, PA). Purified water was obtained using Milli-Q Direct Water Purification System.

**1.2 Purification of commercial FeEDDHA**

Commercial FeEDDHA was obtained and subjected to purification. It is noted that commercial FeEDDHA fertilizer may contain sodium and potassium salts. To begin, an excess amount of commercial FeEDDHA was dissolved in methanol and subsequently filtered. The filtrate obtained was then dried using a rotary evaporator. This procedure was repeated once more to ensure the removal of any residual inorganic particulate matter. The resulting solid was then washed with dichloromethane to eliminate any organic contaminants. The filtrate was subsequently dried, leading to the commencement of recrystallization as the methanol and dichloromethane evaporated. Upon completion, shiny glassy black needles were obtained after drying.


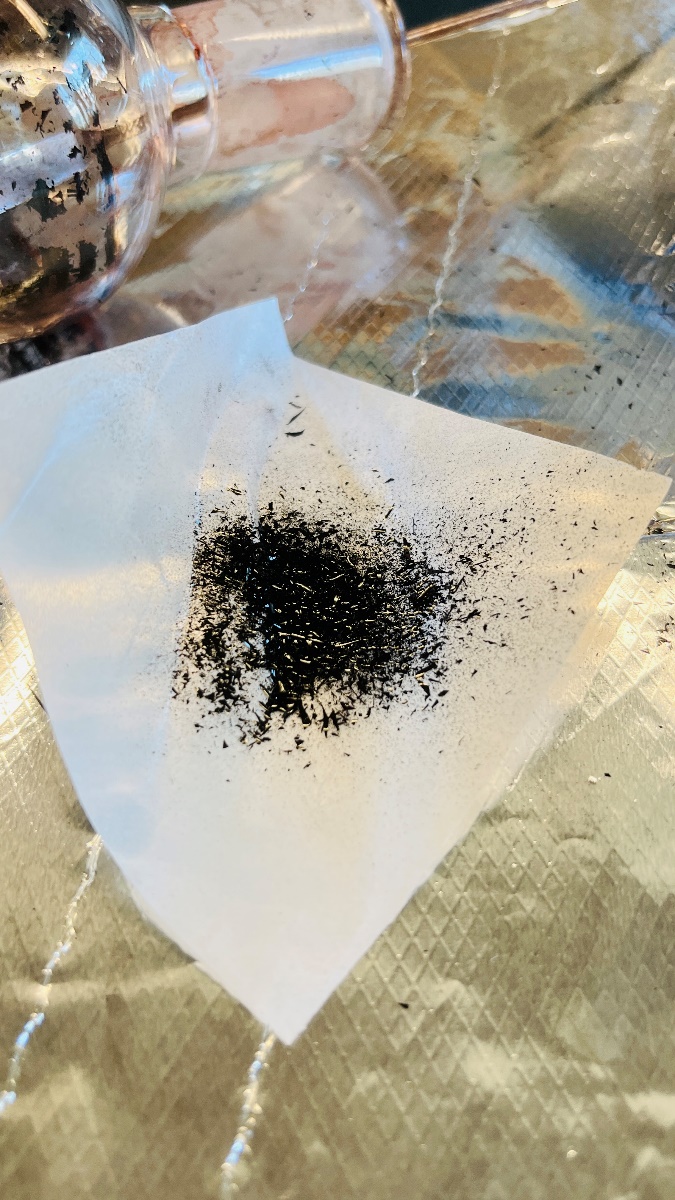


**Figure S1**. Picture of the recrystallized FeEDDHA

The purity of the purified FeEDDHA was confirmed by UV-vis spectroscopy each time a new batch was prepared. The purity of the commercial FeEDDHA was determined to be 72%.

**1.3.** **Crystal** **purity by LCMS**


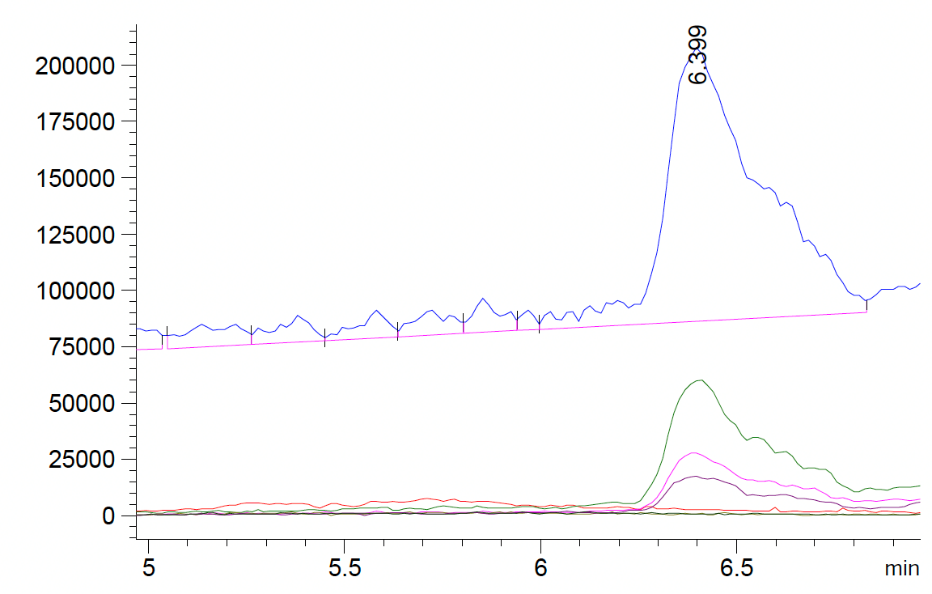


**Figure S2**. Spectrum obtained using LC-MS in negative ion mode with an Agilent 6125B mass spectrometer coupled to an Agilent 1260 Infinity LC system.

**1.4**. **Electrochemical method to determine crystal purity**

It is important to note that this method relies on the assumption of near-unity faradaic efficiency.

The procedure is as follow:

(1) Dissolve 0.2 mmol of the impure FeEDDHA sample in 4 mL of aqueous electrolyte to prepare a 50 mM solution, potentially containing sodium and potassium salts (sample 1).

(2) Conduct electrochemical reduction on Sample 1 for 50% reduction by applying –10 mA for 965 seconds, delivering 0.10 mmol of electrons (Sample 2). This follows the standard procedure for the electrochemical reduction of Fe(III)EDDHA. (sample 2).

(3) Measure the UV-Vis spectra of Sample 1 and Sample 2 after diluting them to 125 µM. Record the peak intensities at 481 nm (I_1_ for Sample 1 and I_2_ for Sample 2).

(4) Calculate the difference in peak intensity at 481 nm after the 50% reduction (I_50,red_ = I_1_ - I_2_)

(5) Prepare a purified FeEDDHA sample (Sample 3) and measure its UV-Vis peak intensity at 481 nm (I_3_).

(6) Determine the purity of the impure sample using the formula:

$$Purity \left( \% \right)=\frac{I_{3}}{I_{50,red}\times2}\times100$$

I_1_: 0.2784 at 481 nm

I_2_: 0.0928 at 481 nm

I_50,red_ = 0.1856

I_3_ = 0.3747 at 481 nm

The calculated purity using the electrochemical method was 100.9%.

**1.5. Instrumentation**

Proton nuclear magnetic resonance (^1^H NMR) spectra were obtained on a Bruker 400 MHz NMR instrument (400 MHz). Chemical shifts for proton and carbon are reported in parts per million (ppm). The following designations are used to describe multiplicities: s (singlet), d (doublet), t (triplet), q (quartet), m (multiplet), br (broad). The mass flow controller (GFCS-014657) and flow meter (GFMS-015835) was purchased from Aalborg Instruments & Controls, INC (Orangeburg, NY) and Avantor Masterflex (Allentown, PA) and was calibrated for CO_2_ by the vendor before use. The CO_2_ FT-IR sensor (GC-0016 for 0–100%, GC-0006 for 0–20% of CO_2_) was purchased from CO2Meter (Ormond Beach, FL) and was calibrated for CO_2_ before use. All cyclic voltammetry (CV) experiments were performed using PARSTAT MC (PMC-2000), from Ametek (Oak Ridge, TN). The UV-Vis Spectrophotometer (Cary 60) was purchased from Agilent (Santa Clara, CA). The pH was measured using a pH probe (Orion PerpHecT ROSS), which was calibrated prior to the experiment.

**1.6. EPR and NMR**

All solutions were prepared using D_2_O (99.9 atom % D, Sigma Aldrich) for EPR and NMR measurements. The solutions with CO_2_ were prepared inside the glove box immediately before EPR and NMR experiments. EPR measurements were performed on a Bruker ELEXSYS E580 spectrometer operated at X-band with a microwave frequency of 9.32 GHz. A capillary with ID 0.8 mm and OD 1.0 mm was used to hold the sample with both ends sealed inside a 4 mm EPR tube. The temperature was reduced gradually to 100 K (1 – 2 hours) using liquid N_2_ tank to avoid the breaking of capillary tube.

^1^H and ^13^C NMR measurements were performed on a Varian-DDR spectrometer on a Bruker Avance Neo spectrometer with a 11.7 T magnet. 90˚ pulse widths were 8.3 µs and 9.0 µs for ^1^H and ^13^C respectively. For quantitative NMR analysis, a 30° pulse width was used with a relaxation delay of 10 s for ^1^H and 300 s for ^13^C to ensure full equilibration between scans. For varying temperature experiments, each sample was equilibrated at the target temperature for 10 min before measurements. Both ^1^H and ^13^C chemical shifts were referenced externally to Tetramethylsilane (TMS) at 0 ppm.

A calibration curve for iron was established by preparing a series of dilutions from an iron standard solution (10,000 µg/mL, Inorganic Ventures). Glycerol (38%) was employed as a cryoprotectant (glassing agent) during the cooling process to 125 K. The double integration of the resulting EPR spectra was used to determine the relationship between the EPR signal area and the iron concentration. This calibration curve was then utilized to estimate the concentration of Fe^3+^ in the samples.

**2. Procedures for electrochemical CO_2_ capture and release**

**2.1. General material information for electrochemical reaction setups in batch**

1. 5 mL H-cell with #9 O-ring was purchased from Adams & Chittenden Scientific Glass Coop (Berkeley, CA).
2. Cation exchange membrane (FKS-30) was purchased from Fuel Cell Store (Bryan, Tx).
3. Graphite felt (G150 AvCarb^Ⓡ^ Soft Graphite Felt) was purchased from Fuel Cell Earth (Stoneham, MA).

**2.2. Procedure for electrochemical reduction of Fe(III)EDDHA solution in batch**

Reactions were carried out with graphite felt (0.5 cm X 0.3 cm X 2 cm was immersed in the solution) cathode and a stainless steel wire anode in 5 mL H-cell with #9 O-ring equipped with cation exchange membrane. In the cathodic chamber, FeEDDHA (87 mg, 0.2 mmol, 50 mM), nicotinamide (488 mg, 4 mmol, 1 M) and potassium nitrate (404 mg, 4 mmol, 1 M) were added into water (4.0 mL). In the anodic chamber equipped with a needle to prevent pressurization was placed potassium nitrate solution (404 mg, 4 mmol, 1 M, 4.0 mL of water). The solution was bubbled with nitrogen for 10 min, after which the electrochemical potential was applied at room temperature by a constant current of –10 mA for 1737 s (0.18 mmol of electrons) to provide 45 mM of Fe(II)EDDHA solution. The solution was monitored by pH meter.

**2.3. Procedure for electrochemical oxidation of Fe(II)EDDHA solution in batch**

Reactions were carried out with graphite felt (0.5 cm X 0.3 cm X 2 cm was immersed in the solution) anode and a stainless steel wire cathode in 5 mL H-cell with #9 O-ring equipped with cation exchange membrane. In the anodic chamber, the Fe(II)EDDHA solution (45 mM, 4 mL) prepared as above was bubbled by 15% CO_2_ for 20 min at a flow rate of 20 mL/min. In the cathodic chamber equipped with a needle to prevent pressurization was placed potassium nitrate solution (404 mg, 4 mmol, 1 M, 4.0 mL of water). The electrochemical potential was applied at room temperature by a constant current of 10 mA for 1737 s (0.18 mmol of electrons). The solution was monitored by pH meter and the gas output from the anodic chamber was measured by a flow meter and FT-IR CO_2_ sensor.

**2.4. Procedure for stability test using UV-vis absorption spectroscopy**

Procedure for reduction of Fe(III)EDDHA was followed with an electrochemical potential of constant current of at –10 mA for 1737 s (0.18 mmol of electrons) to provide 45 mM Fe(II)EDDHA solution. A set of 1 mL of solution in an 8 mL vial was prepared and contacted with 15% CO_2_ (flow rate of 10 mL/min), and pure CO_2_ (flow rate of 10 mL/min) for 20 min. The samples were measured by UV-vis. Each time 25 µL of the samples were collected and diluted with 10 mL water to provide 125 µM solutions that were measured by UV-vis absorption spectroscopy.

**2.5. Procedure for stability test using ^1^H-NMR spectroscopy**

Procedure for reduction of Fe(III)EDDHA was followed with an electrochemical potential of constant current of at –10 mA for 1930 s (0.20 mmol of electrons) to provide 50 mM Fe(II)EDDHA solution. A set of 1 mL of solution in an 8 mL vial was prepared and contacted with 15% CO_2_ (flow rate of 10 mL/min), and pure CO_2_ (flow rate of 10 mL/min) for 20 min. The samples were measured by ^1^H-NMR. Each time 0.5 mL of the samples were collected and added 50 μL deuterated water and the samples were measured by ^1^H-NMR spectroscopy.

**2.6. Procedures for electrochemical capture and release of CO_2_ in continuous flow**

**2.6.1. Procedure for electrochemical capture and release of CO_2_ in cyclic flow from 15% CO_2_**

The 6 mL of 50 mM Fe(III)EDDHA solution in 1 M KNO_3_ and 1 M NA in water was added to a 10 mL three-neck round bottom flask anolyte reservoir equipped with a stir bar. The 6 mL of 1 M KNO_3_ solution was added to a 10 mL round bottom flask catholyte reservoir equipped with a stir bar. Both catholyte and anolyte solutions was continuously bubbled by 15% CO_2_ (balanced by nitrogen) at a flow rate of 5.3 mL/min.

The peristaltic pump equipped with Masterflex^®^ 14 tubing was set to the liquid flow rate of 6 mL/min providing 22 sec of residence time in each 2.2 mL chamber of the flow cell. The electrochemical potential at a constant current mode of –10 mA for 2026 s (0.21 mmol of electrons) was applied to the cell to provide 35 mM of Fe(II)EDDHA with 15 mM of Fe(III)EDDHA (70% state of charge). The solution in the catholyte chamber was emptied and filled with 1.2 mL of 50 mM Fe(II)EDDHA and 4.8 mL of 50 mM Fe(III)EDDHA to provide 10 mM of Fe(II)EDDHA and 40 mM of Fe(III)EDDHA (20% state of charge). The electrochemical potential at a constant current mode of 5 mA for 2894 s (0.15 mmol of electrons) was applied to the cell followed by 1500 s of rest. Each cycle consists –5 mA for 2894 s, rest for 1500 s, 5 mA for 2894 s, and rest for 1500 s. The output gas flow was measured by a flow meter and CO_2_ sensor for over 72 hours.

UV-vis absorption spectra were collected after 72 hours of operation under 15% CO_2_ confirming stability of FeEDDHA under the current conditions.

**3. Setup of FeEDDHA Redox System for the Electrochemical CO_2_ Capture and Release**

**3.1. General material information for electrochemical reaction setups in cyclic flow**

1. Cation exchange membrane (FKS-30) was purchased from Fuel Cell Store (Bryan, Tx).
2. Graphite felt (G150 AvCarb^Ⓡ^ Soft Graphite Felt) was purchased from Fuel Cell Earth (Stoneham, MA).
3. Clear Scratch- and UV-Resistant Cast Acrylic Sheet (7/16) was purchased from McMaster-Carr (Aurora, OH).
4. Titanium foil, 0.025mm (0.001in) thick, 99.94% (metals basis) was purchased from Thermo Scientific (Waltham, MA).
5. Silicone rubber sheet 0.125in thick was purchased from Rogers corporation (Chandler, AZ).
6. Peristaltic tubing (Masterflex Versilon Chemical (06475)–14) was purchased from Masterflex (Radnor, PA).
7. Tygon^Ⓡ^ tubing (ID 1/16in, OD 3/16in) was purchased from McMaster-Carr (Aurora, OH).
8. The peristaltic pump (Masterflex L/S) was purchased from Masterflex (Radnor, PA).

As shown in Figure 4a and S1, a cyclic flow cell with graphite felt anode and cathode with a cation exchange membrane was connected to a potentiostat. The flow cell was connected to Tygon^Ⓡ^ tubing (ID 1/16 in, OD 3/16 in) at a liquid inlet (bottom) and outlet (top) of anodic and cathodic chambers (thru-hole 1/16 in). A peristaltic pump was used to deliver the electrolyte solution from the catholyte and anolyte reservoirs. The distal end of the tubing system from the cathodic chamber was connected to the catholyte reservoir and the tubing from the anodic chamber to the anolyte reservoir. The 15% CO_2_ gas cylinder was connected to tubing and its stream was metered by a mass flow controller (MFC). The 15% CO_2_ stream was introduced to the catholyte and anolyte solutions at a flow rate of 5.3 mL/min. FT-IR CO_2_ sensor and a flow meter were installed to monitor the output gas during cyclic flow operation from the headspace of catholyte and anolyte chambers.


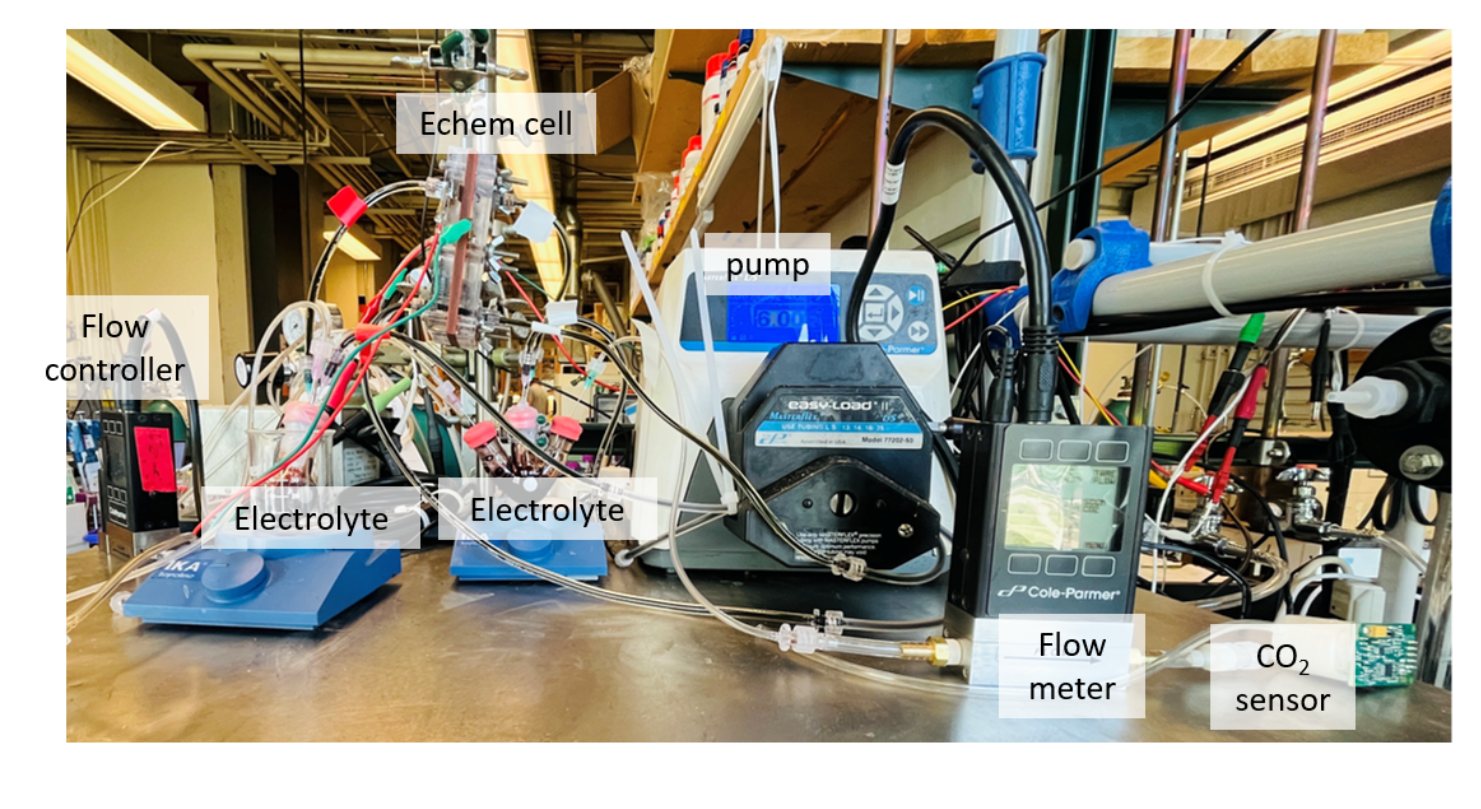


**Figure S3**. Photograph of setup for electrochemical capture and release of CO_2_ in cyclic flow.

**4. Energy** **Calculation**

The energy requirement using 15% CO_2_ in cyclic flow is:

Average of cell potential under 15% CO_2_ during operation for 29 cycles (*E*_cell,ave_)= 0.944 V

*Energy_ave_* = *E*_cell,ave_ × 96.49 kJ/mol·eV × (*ε_CO2_*)^-1^ = 63.7 kJ_e_/mole of CO_2_

Where the average value of *ε_CO2_* for electron utilization is 1.43 in cyclic flow for 29 cycles obtained in Figure 7e.

The minimum energy requirement using 15% CO_2_ during operation in cyclic flow is:

Cell potential under 15% CO_2_ for cycle 1 (*E*_cell,min_)= 0.335 V

*Energy_min_* = *E*_cell,min_ × 96.49 kJ/mol·eV × (*ε_CO2_*)^-1^ = 22.6 kJ_e_/mole of CO_2_

Where the value of *ε_CO2_* for electron utilization is 1.42 in cyclic flow for cycle 1 obtained in Figure 7e.

**5. Additional UV-vis Absorption Spectroscopy Data**

| FeEDDHA conc. (µM) | Intensity at 481 nm |
| --- | --- |
| 250 | 0.744004 |
| 125 | 0.375031 |
| 62.5 | 0.190558 |
| 31.25 | 0.094601 |
| 15.6 | 0.048291 |


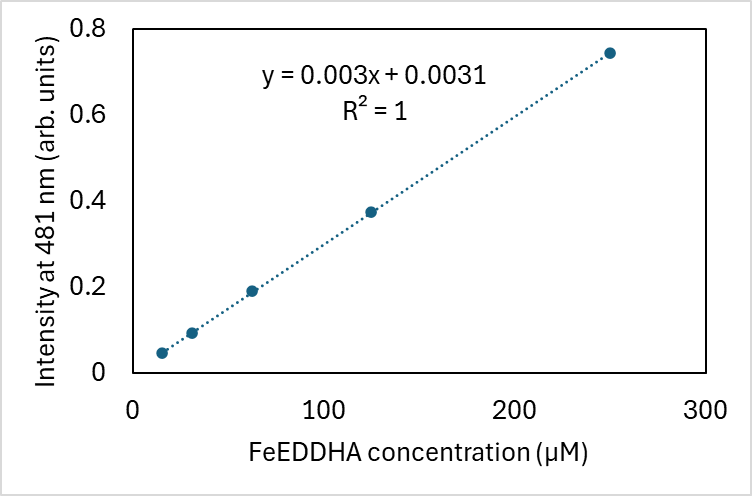


**Figure S4**. UV vis calibration curve of Fe(III)EDDHA concentration. All samples were diluted in phosphate buffer (pH 7.4) prior to measurement.

**
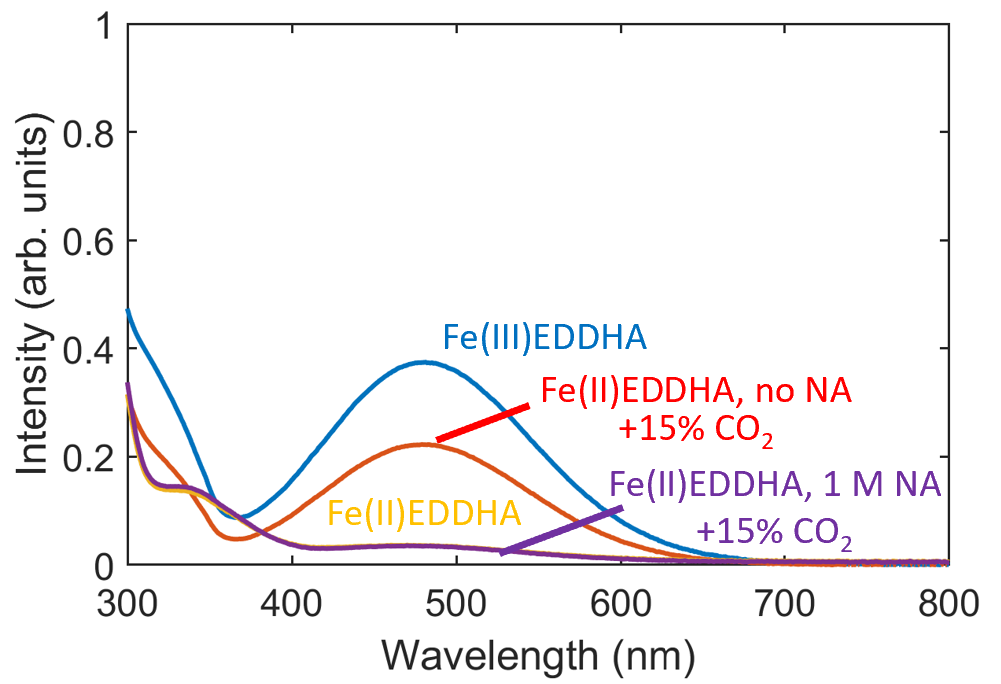
**

**Figure S5.** UV-vis spectra for the CO_2_ sensitivity test. The Fe(II)EDDHA solutions in the absence and presence of 1 M NA (yellow curve, 50 mM, 4 mL) were bubbled with 15% CO_2_ for 20 min at a flow rate of 10 mL/min in the absence of NA (red curve), and in the presence of 1 M NA (purple curve). Blue curve represents UV-vis spectra of Fe(III)EDDHA.

**
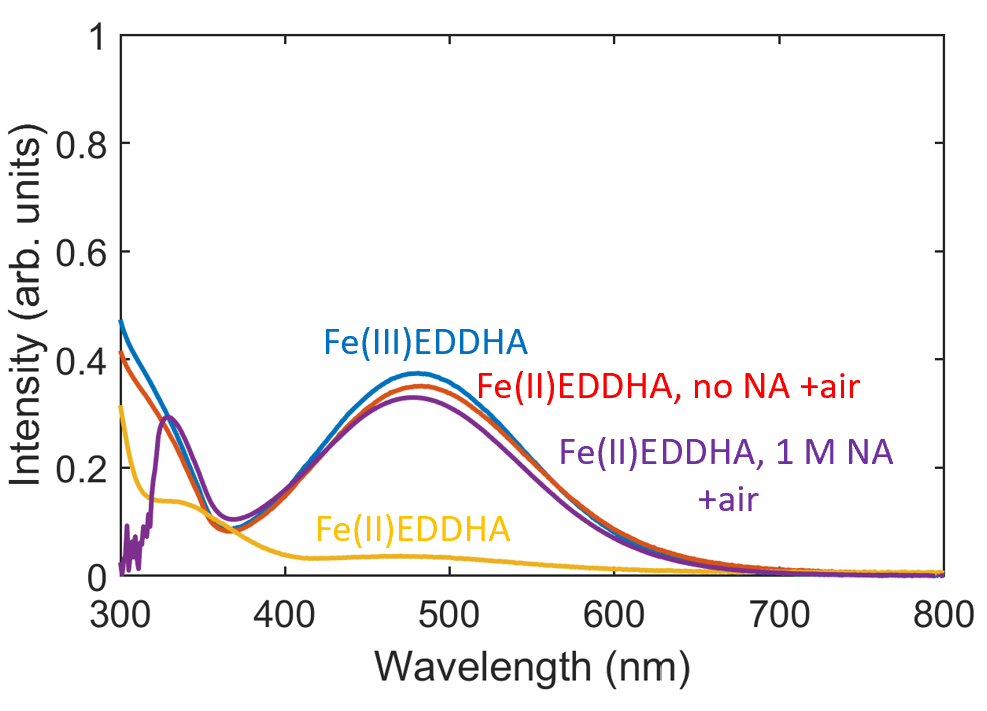
**

**Figure S6.** UV-vis spectra for the air sensitivity test. The Fe(II)EDDHA solutions in the absence and presence of 1 M NA (yellow curve, 50 mM, 4 mL) were bubbled with air for 20 min at a flow rate of 10 mL/min in the absence of NA (red curve), and in the presence of 1 M NA (purple curve). Blue curve represents UV-vis spectra of Fe(III)EDDHA.


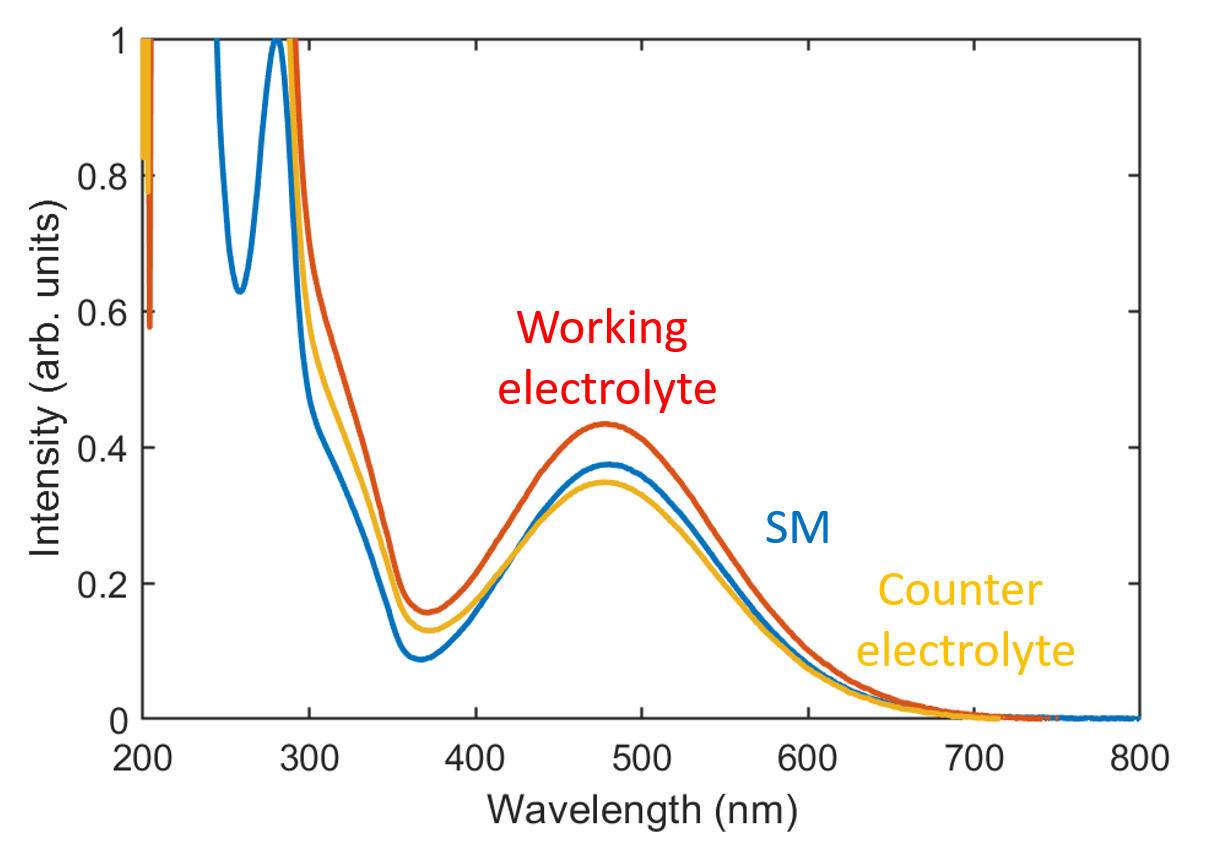


**Figure S7.** UV-vis spectra after cyclic flow experiment for 29 cycles. The solutions were exposed to air to be oxidized and measured. Red curve represents sample from the working electrolyte and the yellow curve from the counter electrolyte.

**6. Additional NMR/EPR Spectroscopy Data**

**
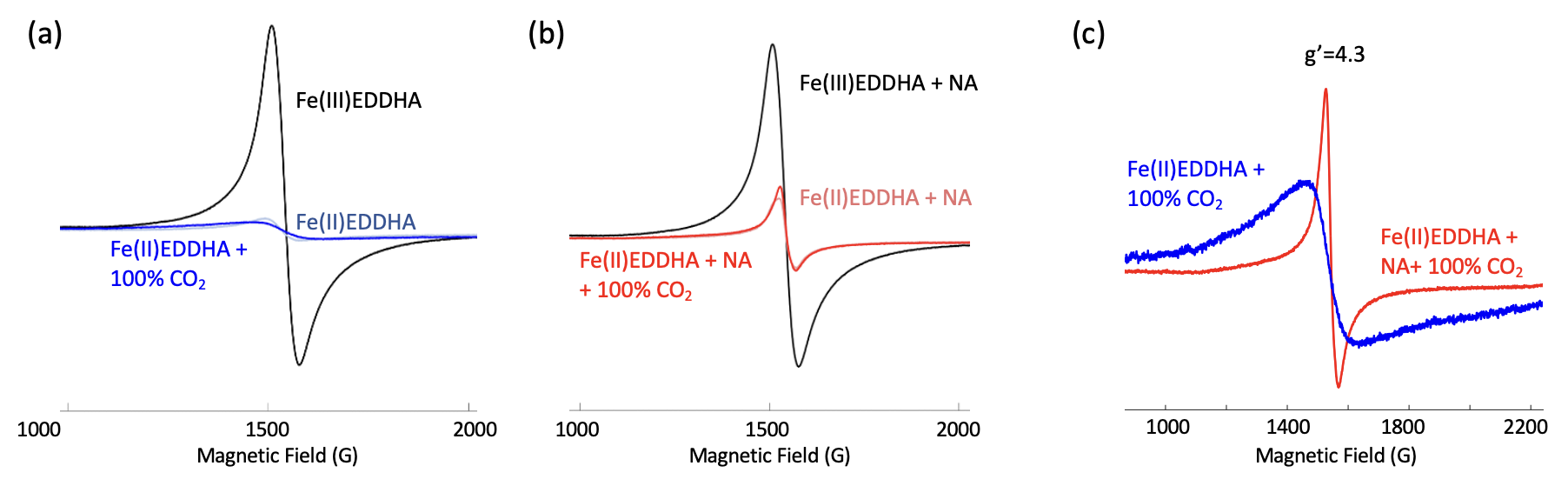
**

**Figure S8.** (a) X-band EPR spectra of 50 mM Fe-EDDHA in 1 M KNO_3_ aqueous solutions without NA collected at 100 K. The black, light blue, and blue curves represent the pristine Fe(III)-EDDHA, Fe(II)-EDDHA (after electrochemical reduction) and Fe(II)-EDDHA with 100% CO_2_. The g’=4.3 EPR signals of Fe(III) are located at ~1600 G at the X-band EPR, while Fe(II) in the solutions is not detectable in EPR. (b) X-band EPR spectra of 50 mM Fe-EDDHA in 1 M KNO_3_ aqueous solutions with 1 M NA collected at 100 K. The black, pink, and red curves represent the pristine Fe(III)-EDDHA, Fe(II)-EDDHA (after electrochemical reduction) and Fe(II)-EDDHA with 100% CO_2_. The g’=4.3 EPR signals of Fe(III) are located at ~1600 G at the X-band EPR, while Fe(II) in the solutions is not detectable in EPR. (c) X-band EPR spectra of 50 mM reduced Fe(II)-EDDHA in 1 M KNO_3_ aqueous solutions after bubbling with 100% CO_2_ for 2 minutes in the absence of NA (blue) and with 1 M NA (red) collected at 100 K. Note here the g’=4.3 EPR signal is from Fe(III) because low-spin Fe(II) is not detectable in EPR.


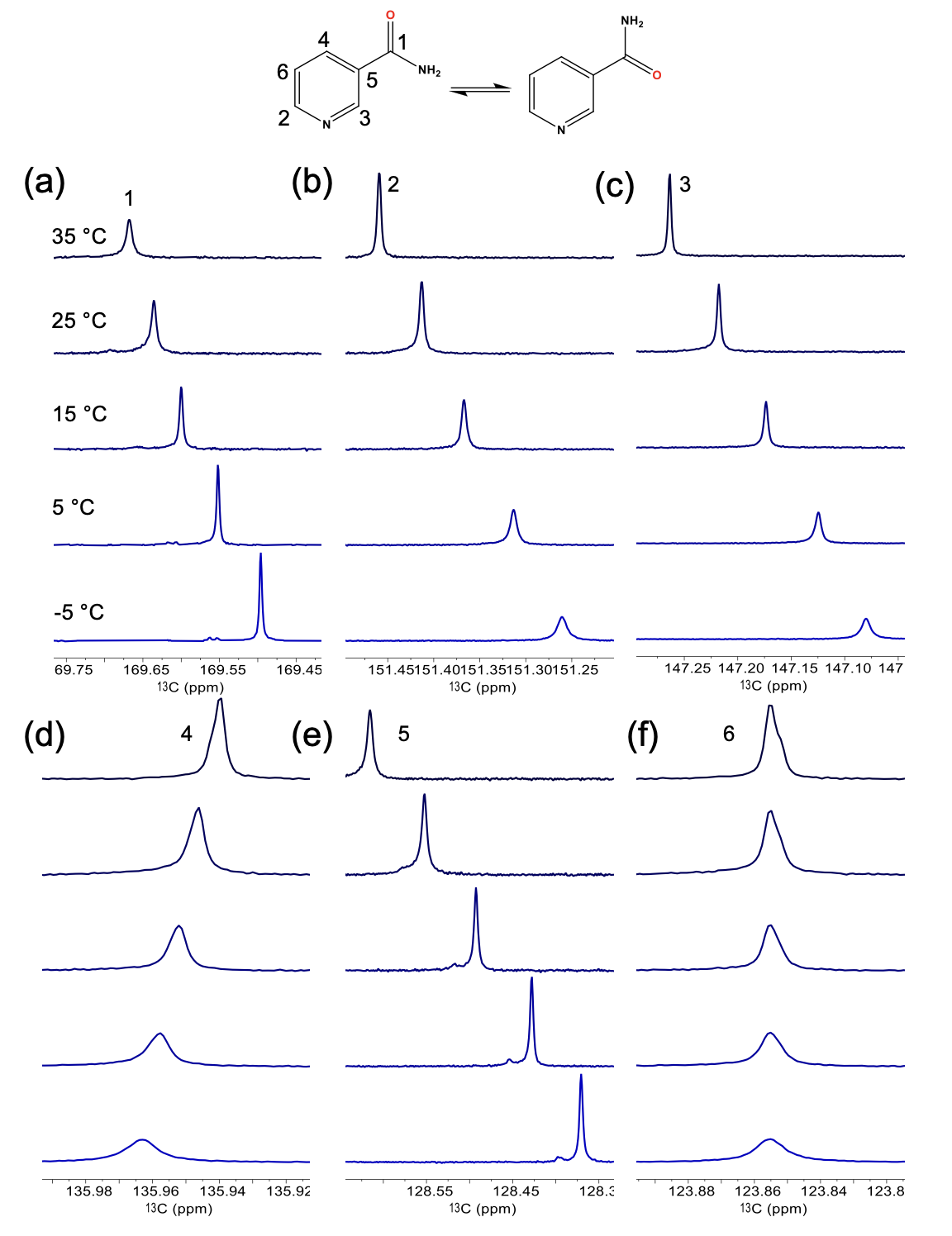


**Figure S9**. ^13^C NMR spectra of 1 M NA in D_2_O at varying temperatures demonstrating the effects of

chemical exchange on NMR spectra.


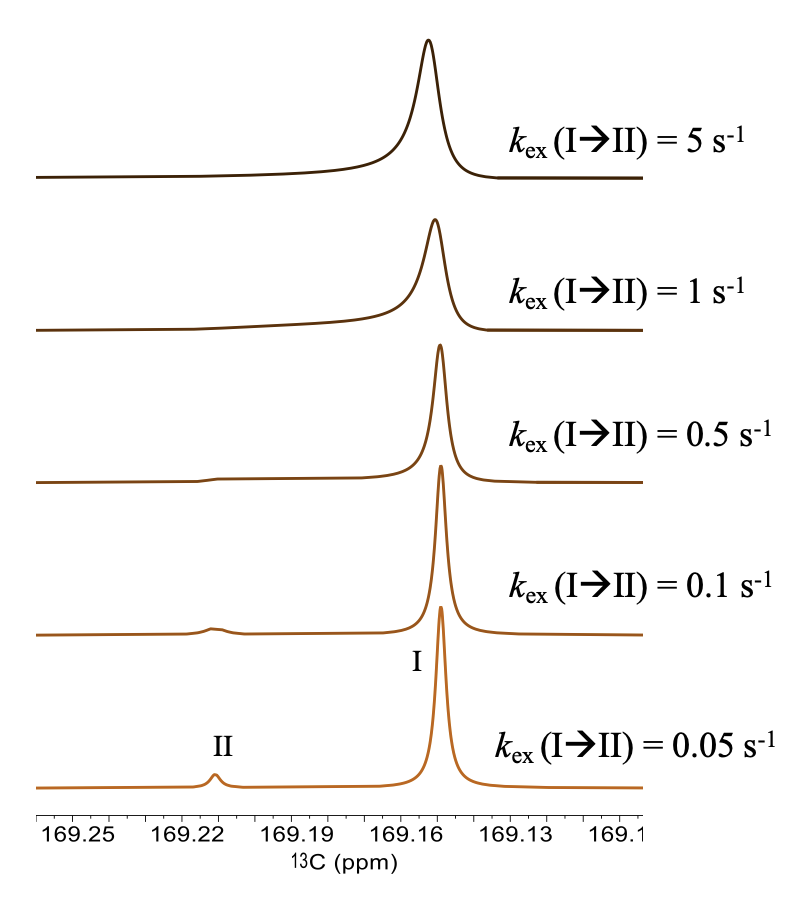


**Figure S10**. Simulated ^13^C spectra using two-site exchange model, with the fraction of site I ~ 93% and site

II ~ 7% and the exchange rates of site I to site II varying between 0.05 s^-1^ and 5 s^-1^.


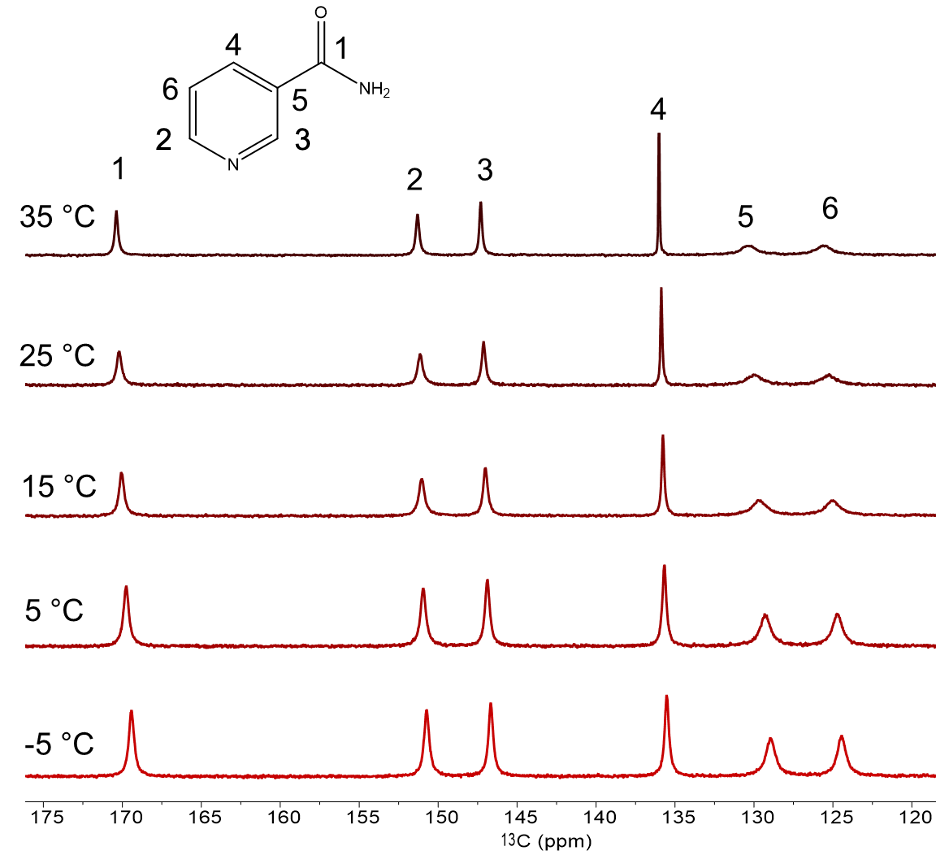


**Figure S11**. ^13^C NMR spectra of Fe(II)EDDHA + 1 M NA at varying temperatures.

The concentrations of bicarbonate/carbonate and dissolved CO₂ were estimated using a calibration curve generated from a series of ¹³C-enriched NaHCO₃ solutions ranging from 1 mM to 100 mM. For solutions containing NA, the relative integrals of the ¹³C NMR signals from bicarbonate/carbonate/CO₂ and NA carbons can also be used to quantify their concentrations. For example, in the ¹³C NMR spectrum of Fe(II)-EDDHA with 1.0 M NA bubbled with 99% ¹³C-enriched CO₂ at –5 °C (Figure S12), line fitting of the peaks between 142 and 176 ppm yields an integration ratio of approximately 2.75:1 for dissolved bicarbonate/carbonate relative to NA. Given the natural abundance of ¹³C in NA (1.1%) and the 99% ¹³C enrichment of CO₂, and knowing that the NA concentration is 1.0 M, the estimated concentration of dissolved bicarbonate/carbonate is ~30.6 mM. Both methods yield consistent results within an accuracy of ±5%.


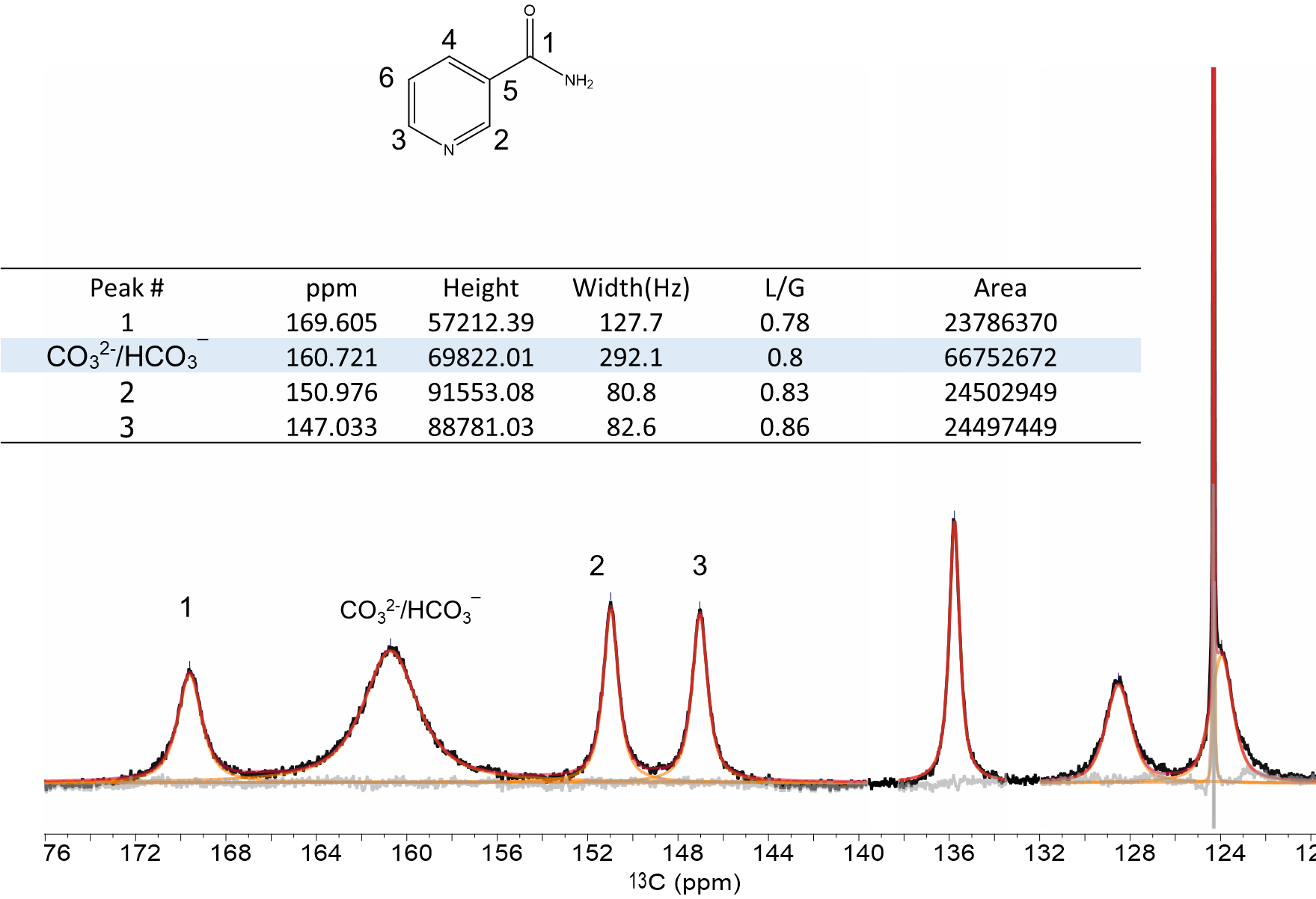


**Figure S12**. Line fitting (deconvolution) of ^13^C signals reveals the concentrations of absorbed carbonate/bicarbonate and CO_2_ based on the concentration of NA and the abundance difference of ^13^C in NA and CO_2_.

For solutions containing NA, the concentrations of carbonate/bicarbonate and CO₂ can be estimated at different temperatures by comparing their ¹³C signal integrals relative to that of NA. For solutions without NA, the relative fraction of carbonate/bicarbonate to CO₂ can be determined from their integration ratios; so if assuming that the total concentration of carbonate, bicarbonate, and CO₂ remains constant between –5 °C and 25 °C, the concentration of each species can be estimated accordingly. In both Fe(II)/NA/CO_2_ and Fe(II)/CO_2_ solutions, the concentration of bicarbonate/carbonate slightly increases as temperature decreases, while the concentration of dissolved CO_2_ correspondingly decreases (Figure S13).

**Figure S13**. Concentration of bicarbonate/carbonate and dissolved CO_2_ in the solutions of Fe(II)-EDDHA + 1 M NA and Fe(II)-EDDHA without NA as a function of temperature.

**Table S1**. The concentration of bicarbonate/carbonate and dissolved CO_2_ estimated from quantitative ^13^C NMR.

|  | Bicarbonate/carbonate (mM) | Dissolved CO_2_ (mM) |
| --- | --- | --- |
| NA/CO_2_ | 2.2 | 14.1 |
| Fe(III)/CO_2_ | 2.1 | 1.8 |
| Fe(II)/CO_2_ | 9.1 | 4.0 |
| Fe(II)/NA/CO_2_ | 23.1 | 12.9 |

The addition of 1 M NA in the 50 mM Fe(II)EDDHA (with 100 mM OH^-^) increases the saturation concentration of carbonate/bicarbonate significantly from 7.9 mM to 20.8 mM, while the bicarbonate concentration reaches only 2 mM at 1 M NA in the absence of Fe(II)EDDHA (Figure S5), indicating that bicarbonate/carbonate may form loose associates with the Fe(II)/NA complex, which increases the adsorption of CO_2_ and carbonate/bicarbonate in the solutions with NA.


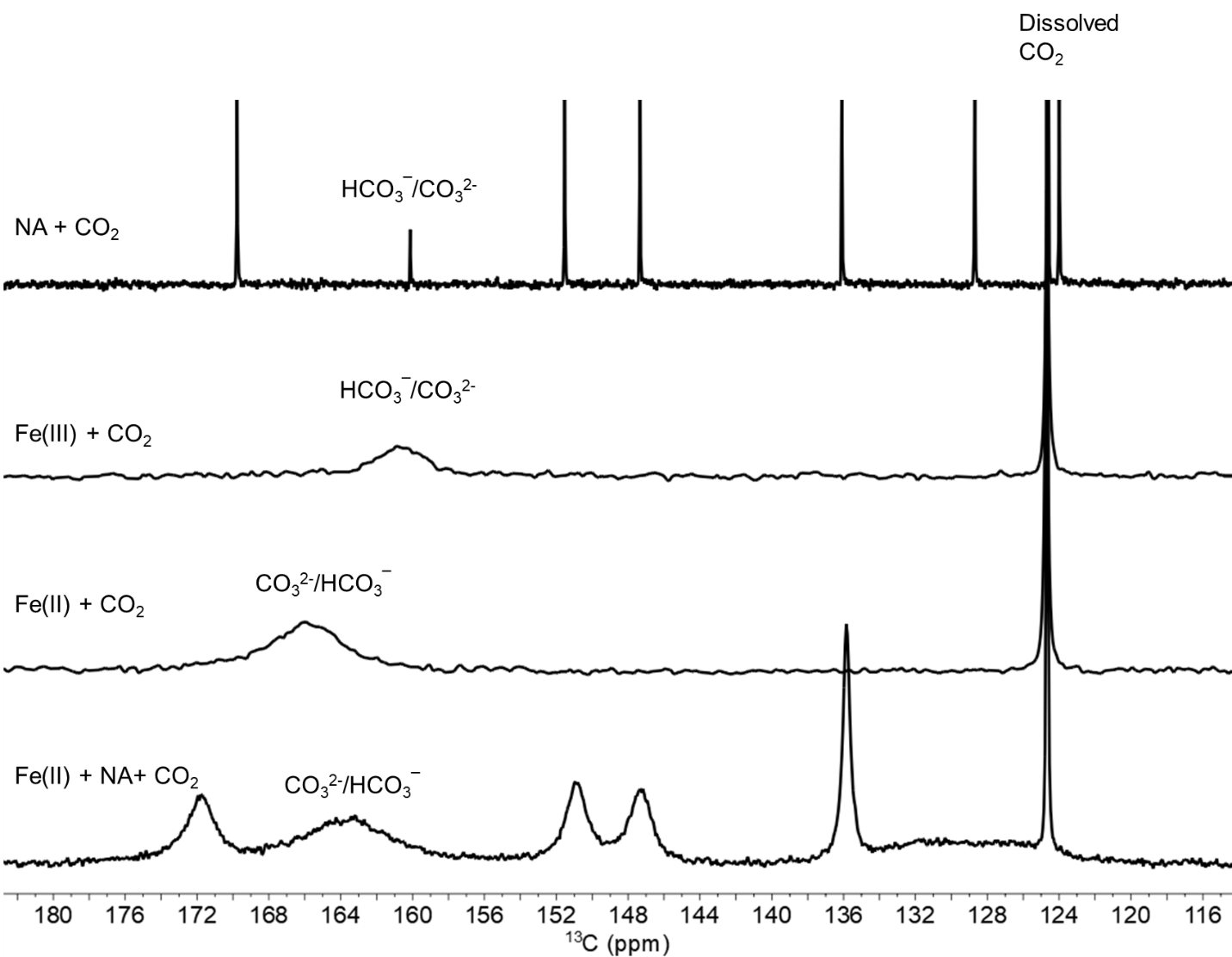


**Figure S14**. ^13^C NMR spectra of 1 M NA + CO_2_, 50 mM Fe(III)EDDHA + CO_2_, 50 mM Fe(II)EDDHA + CO_2_, and 50 mM Fe(II)EDDHA + 1 M NA + CO_2_.

**7. Additional Cyclic Flow Experiment Results**


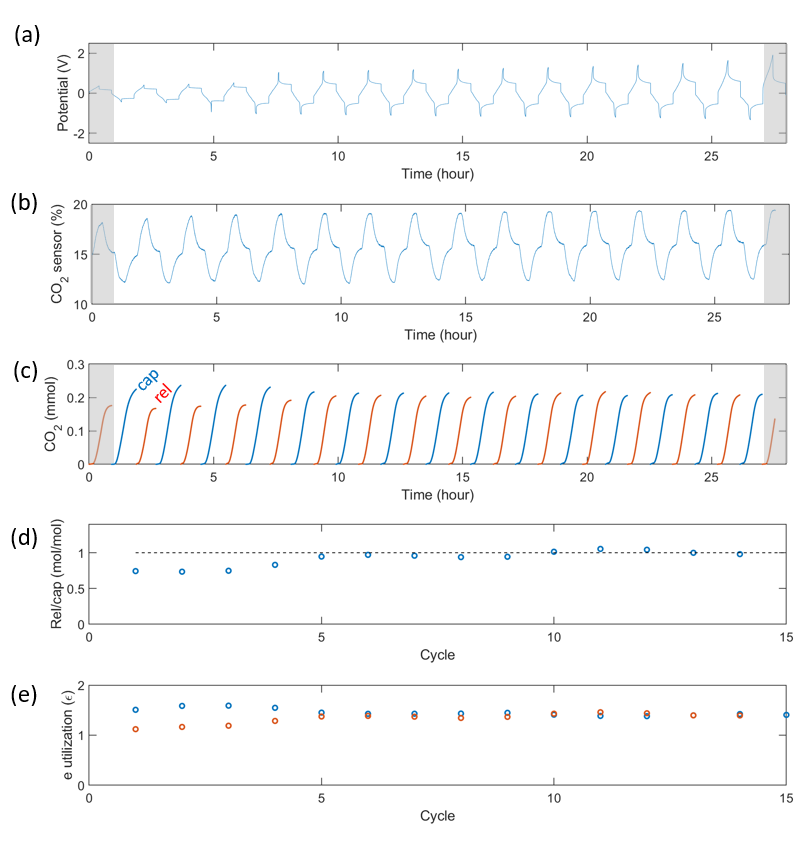


**Figure S15.** Additional multiple cycles demonstration of CO_2_ capture and release using FeEDDHA redox system with assembly of cell components in a glovebox for the extensive air removal. Operations with the constant current mode at 10 mA with 50% capacity usage. Minimum energy requirement was 12.7 kJ_e_/mol and the average energy requirement over 14 cycles were 23.9 kJ_e_/mol. (a) Potential for 14 cycles. (b) CO_2_ sensor (c) Captured (blue curve) and released (red curve) CO_2_ amount over time using 15% CO_2_ (d) Ratio of released CO_2_ amount over captured amount (e) Electron utilization (captured: blue, released: red).

**8. Additional Mass Spectroscopy Experiment**

We performed MS analysis in the negative ion mode to investigate the coordination stoichiometry of NA with Fe(II)EDDHA. The MS spectrum revealed a prominent peak at m/z = 654.38, which corresponds to the complex [FeEDDHA + 2 NA]^3^⁻. This matches the expected molecular weight calculated as follows:

FeEDDHA: C₁₈H₁₄FeN₂O₆, MW = 410.02 g/mol

2 NA: 2 × C₆H₆N₂O, MW = 2 × 122.05 = 244.10 g/mol

Total: 410.02 + 244.10 = 654.12 g/mol

The observed m/z value of 654.38 aligns closely with the calculated mass, providing evidence for the coordination of two NA molecules to a single Fe(II)EDDHA center.

**9. Determination of pKa Values for Fe-EDDHA via Titration Experiments**


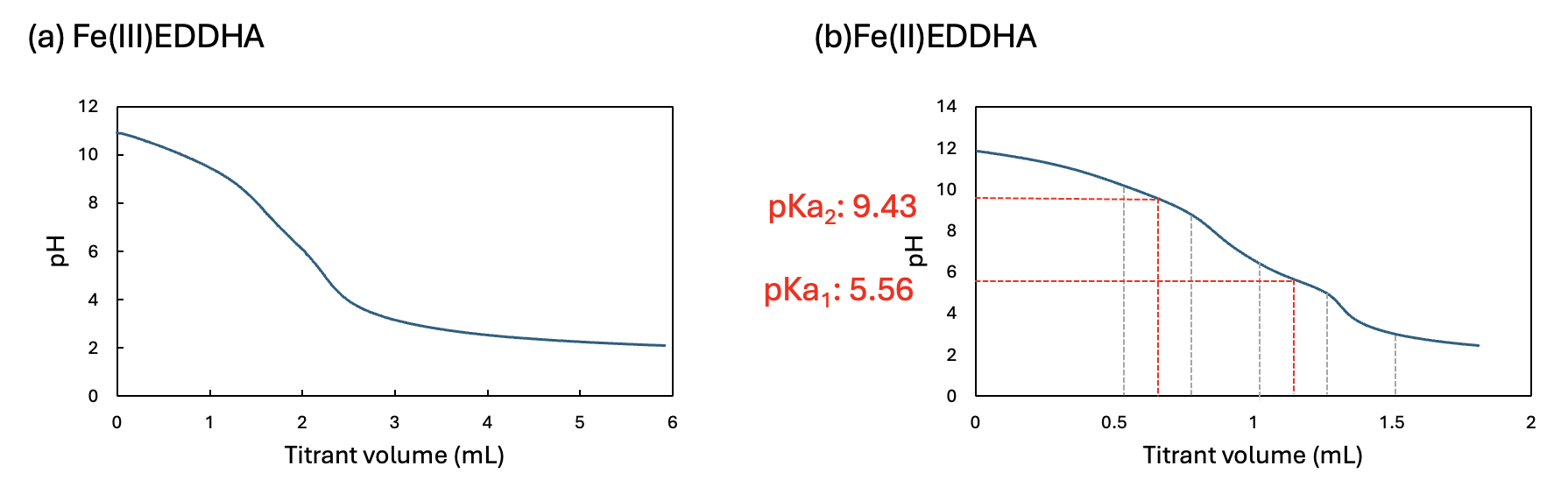
**Figure S16**. Titration of pKa values for Fe-EDDHA. (a) Titration of 30 mM Fe(III)-EDDHA in 5 mL of water. Titrant: 50 mM HCl, injection rate: 0.3 mL/min. (b) Titration of 95% reduced Fe(II)-EDDHA (28.5 mM) in 1 mL of 1 M KCl solution. Initial pH was adjusted by adding 0.3 M NaOH. Titrant: 50 mM HCl, injection rate: 0.1 mL/min.

**10.** **Bjerrum plot under 15% CO_2_**

1. Redox-triggered basification (Fe(III) → Fe(II)).

Reduction of Fe(III)-EDDHA releases OH⁻ (via protonation of hemi-labile phenolates, pK_a1_ = 5.56, pK_a2_ = 9.43), raising the local pH to ~9. At this pH the Bjerrum plot shows >90 % of dissolved inorganic carbon (DIC) exists as HCO_3_⁻.

1. Equilibrium-driven CO_2_ capture.

The pH rise shifts the gas/liquid equilibrium toward dissolution of CO_2_ and conversion to HCO_3_⁻, increasing total DIC.

1. Redox-triggered acidification (Fe(II) → Fe(III)).

Re-oxidation re-coordinates the phenolates, releases H⁺, and lowers the pH to ~6.8.

1. Equilibrium-driven CO_2_ release.

The lower pH shifts the equilibrium back toward molecular CO_2_, decreasing DIC and liberating CO_2_ gas.


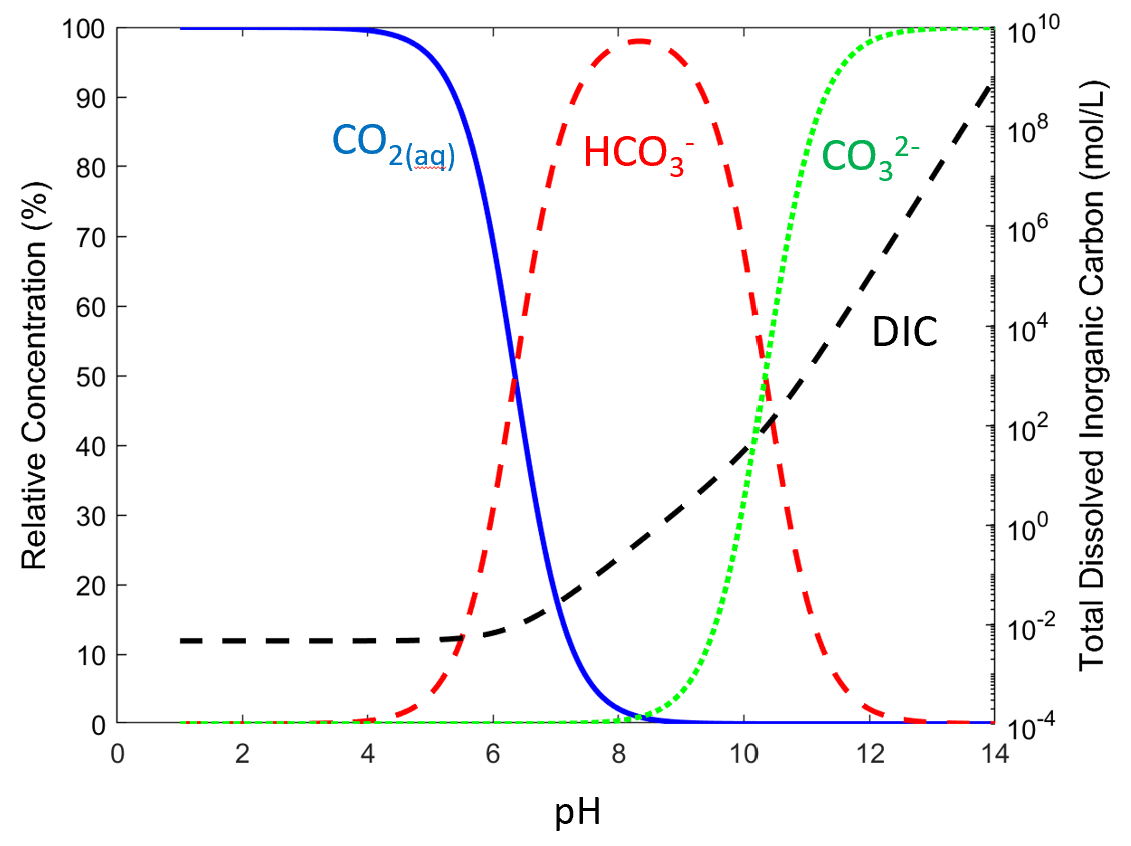


**Figure S17**. Bjerrum plot under 15% CO_2_.

**11. Summary of Selected Electrochemical Carbon Capture Systems**

**Table S2.** Summary of selected electrochemical carbon capture systems using redox-active molecules

|  | **Description** | **Min. work of separation (kJ_e_/mol)** | **Demonstrated work**  **(kJ_e_/mol)** | **Remarks** |
| --- | --- | --- | --- | --- |
| FeEDDHA  (This work) | pH swing in aqueous solution | Est. 21.5^a^ | 22-64 | Electron utilization of 1.43 achieved; 15% CO_2_ in and 17% CO_2_ out |
| 1-AP nitrate^[1]^ | pH swing in aqueous solution | Est. ~64 | 101 | Homogeneous aqueous solution;  Potential DAC applicability;  15% CO_2_ in and 100% CO_2_ out |
| 9,10-phenanthrenequinone^[2]^ |  | Est. ~34 | ~56 (15% CO_2_) | O_2_ sensitivity generally limits current DAC applicability;  Requires organic solvent or ionic liquid |
| 2,6-di-*tert*-butyl-1,4-benzoquinone^[3]^ |  | Est. ~61 | ~ 97 | <1% CO_2_ in and 100% CO_2_ out |
| N-propyl-4,4′-bipyridinium^[4]^ |  | Est. ~19 | N/A | Acetonitrile  solvent |
| 4,4′-bipyridine^[5]^ |  | Est. ~67 | N/A | 1,2-dichloroethane solvent |
| Co(III) complex^[6]^ | CO_2_ sorbent: cyclopentadienyl indenyl Co(III) complex | Est. ~34 | N/A | CO_2_ reduction;  Irreversible decomposition |
| benzyldisulfide^[7]^ | CO_2_ sorbent: benzylthiolate | Est. ~193 | N/A | Demonstrated in DMF |
| 1,8-ESP^[8]^ | pH swing in aqueous solution |  | 36-55 | High concentration (0.8 M); Asymmetric electrochemical cell; 10% CO_2_ in and 100% CO_2_ out |
| FMN/FMNH_2_^[9]^ | pH swing in aqueous solution |  | 9.8 | Biological Redox Proton Carrier; 15% CO_2_ in and 100% CO_2_ out |
| DHPS^[10]^ | pH swing in aqueous solution |  | 21.6 | 15% CO_2_ in and 100% CO_2_ out |
| AzPy^[11]^ |  | 16 | 120 | Demonstrated in DMSO; 18.5% CO_2_ and 3% O_2_ in and 24% CO_2_ out |
| Isoindigo^[12]^ |  | 9.8-27.6 | 127 (10% CO_2_) | Demonstrated in DMSO; 10% CO_2_ in and 14% CO_2_ out |
| Indigo^[13]^ |  |  | 237 (19% CO_2_, 5% O_2_) | Demonstrated in DMSO; 15% CO_2_ in and 30% CO_2_ out |

^a^Estimated from the peak potential difference (reductive peak potential under N_2_ and oxidative peak potential under CO_2_) in Figure 2a in the main text, assuming an ideal electron utilization of 2.

**Table S3**. Summary of selected other benchmark electrochemical carbon capture systems

|  | **Description** | **Min. work of separation (kJ_e_/mol)** | **Demonstrated work**  **(kJ_e_/mol)** | **Remarks** |
| --- | --- | --- | --- | --- |
| FeEDDHA  (This work) | pH swing in aqueous solution | Est. 21.5^a^ | 22-64 | Electron utilization of 1.43 achieved; 15% CO_2_ in and 17% CO_2_ out |
| EMAR^[14,15]^ | CO_2_ sorbent: Ethylene diamine;  Electrochemical swing between Cu(II) and Cu(0) | 16 | ~35 | Repetitive polarity switches; Aqueous solution; 15% CO_2_ in and 100% CO_2_ out |
| Polyanthraquinone^[16]^ |  | 40-90 | 43  at 60% bed utilization | Same benefits and downsides as traditional quinone |
| PCET: MnO_2_^[17,18]^ | pH swing  K_2_CO_3_ capture solution | 33 | N/A | Same benefits and downsides as traditional K_2_CO_3_ |
| BPMED | Water splitting | 120-240 | 210-430 | Split water at BPMs, high cost of membrane, membrane decomposition |
| MCDI^[19]^ | Capacitive | > 5 | 40-50 (15% CO_2_) |  |

^a^Estimated from the peak potential difference (reductive peak potential under N_2_ and oxidative peak potential under CO_2_) in Figure 2a in the main text, assuming an ideal electron utilization of 2.

Our estimated energy consumption is also comparable to the energy demands of temperature-swing processes, which typically require 110-210 kJ thermal per mole of CO_2_ (equivalent to 33-63 kJ_e_/mol with Carnot efficiency of 0.3) for flue gas capture.^[20–23]^ While temperature-swing processes are well-established, our system offers additional advantages in isothermal operation, modularity, and plug-and-play mode of operation as highlighted in the introduction of our manuscript, with energy demands that are within a comparable range.

The field of carbon capture is striving to achieve energy demands as low as 1.8 GJ per ton of CO_2_ (equivalent to 79.2 kJ/mol CO_2_ and 24 kJ_e_/mol CO_2_ with Carnot efficiency of 0.3) for post-combustion carbon capture (DoE Report: DOE/NETL-2009/1366). Our results, although higher than this target, represent a significant step forward in the development of energy-efficient electrochemical systems, especially considering the stability and robustness demonstrated over multiple cycles.

Thermodynamic minimum energy for CO_2_ separation and second-law efficiencies are well-established in the literature. These studies quantify the theoretical energy requirements for CO₂ separation, based on gas composition and ideal separation scenarios, highlighting inherent energy limitations.^[24,25]^

1. **References**

[1] H. Seo, M. Rahimi, T. A. Hatton, *J. Am. Chem. Soc.* 2022, *144*, 2164–2170.

[2] M. B. Mizen, M. S. Wrighton, *Journal of The Electrochemical Society* 1989, *136*, 941–946.

[3] P. Scovazzo, J. Poshusta, D. DuBois, C. Koval, R. Noble, *J. Electrochem. Soc.* 2003, *150*, D91.

[4] H. Ishida, T. Ohba, T. Yamaguchi, K. Ohkubo, *Chem. Lett.* 1994, *23*, 905–908.

[5] R. Ranjan, J. Olson, P. Singh, E. D. Lorance, D. A. Buttry, I. R. Gould, *J. Phys. Chem. Lett.* 2015, *6*, 4943–4946.

[6] D. L. DuBOIS, A. Miedaner, W. Bell, J. C. Smart, in *Electrochemical and Electrocatalytic Reactions of Carbon Dioxide*, Elsevier, 1993, pp. 94–117.

[7] P. Singh, J. H. Rheinhardt, J. Z. Olson, P. Tarakeshwar, V. Mujica, D. A. Buttry, *J. Am. Chem. Soc.* 2017, *139*, 1033–1036.

[8] S. Pang, S. Jin, F. Yang, M. Alberts, L. Li, D. Xi, R. G. Gordon, P. Wang, M. J. Aziz, Y. Ji, *Nat Energy* 2023, *8*, 1126–1136.

[9] H. Xie, W. Jiang, T. Liu, Y. Wu, Y. Wang, B. Chen, D. Niu, B. Liang, *Cell Reports Physical Science* 2020, *1*, 100046.

[10] H. Xie, Y. Wu, T. Liu, F. Wang, B. Chen, B. Liang, *Applied Energy* 2020, *259*, 114119.

[11] X. Li, X. Zhao, Y. Liu, T. A. Hatton, Y. Liu, *Nat Energy* 2022, *7*, 1065–1075.

[12] X. Li, X. Zhao, L. Zhang, A. Mathur, Y. Xu, Z. Fang, L. Gu, Y. Liu, Y. Liu, *Nat Commun* 2024, *15*, 1175.

[13] K. N. Jayarapu, A. Mathur, X. Li, A. Liu, L. Zhang, J. Kim, H. Kim, S. K. Kuk, Y. Liu, *Adv Funct Materials* 2024, 2402355.

[14] M. C. Stern, F. Simeon, H. Herzog, T. A. Hatton, *Energy Environ. Sci.* 2013, *6*, 2505.

[15] M. Wang, S. Hariharan, R. A. Shaw, T. A. Hatton, *International Journal of Greenhouse Gas Control* 2019, *82*, 48–58.

[16] S. Voskian, T. A. Hatton, *Energy Environ. Sci.* 2019, *12*, 3530–3547.

[17] M. Rahimi, G. Catalini, S. Hariharan, M. Wang, M. Puccini, T. A. Hatton, *Cell Reports Physical Science* 2020, *1*, 100033.

[18] M. Rahimi, G. Catalini, M. Puccini, T. A. Hatton, *RSC Adv.* 2020, *10*, 16832–16843.

[19] L. Legrand, O. Schaetzle, R. C. F. De Kler, H. V. M. Hamelers, *Environ. Sci. Technol.* 2018, *52*, 9478–9485.

[20] T. N. G. Borhani, A. Azarpour, V. Akbari, S. R. Wan Alwi, Z. A. Manan, *International Journal of Greenhouse Gas Control* 2015, *41*, 142–162.

[21] R. T. J. Porter, M. Fairweather, C. Kolster, N. Mac Dowell, N. Shah, R. M. Woolley, *International Journal of Greenhouse Gas Control* 2017, *57*, 185–195.

[22] M. E. Boot-Handford, J. C. Abanades, E. J. Anthony, M. J. Blunt, S. Brandani, N. Mac Dowell, J. R. Fernández, M.-C. Ferrari, R. Gross, J. P. Hallett, R. S. Haszeldine, P. Heptonstall, A. Lyngfelt, Z. Makuch, E. Mangano, R. T. J. Porter, M. Pourkashanian, G. T. Rochelle, N. Shah, J. G. Yao, P. S. Fennell, *Energy Environ. Sci.* 2014, *7*, 130–189.

[23] K. A. Mumford, Y. Wu, K. H. Smith, G. W. Stevens, *Front. Chem. Sci. Eng.* 2015, *9*, 125–141.

[24] K. Z. House, A. C. Baclig, M. Ranjan, E. A. van Nierop, J. Wilcox, H. J. Herzog, *Proceedings of the National Academy of Sciences* 2011, *108*, 20428–20433.

[25] L. Matthews, W. Lipiński, *Energy* 2012, *45*, 900–907.
